# Supplementary material for: Growth strategy of microbes on mixed carbon sources
Source: Nat Commun. 2019 Mar 20;10:1279. doi: 10.1038/s41467-019-09261-3 (PMC6427025; doi:10.1038/s41467-019-09261-3)
Supplement: Supplementary file 1 — Supplementary Information [file 41467_2019_9261_MOESM1_ESM.pdf]

# Growth strategy of microbes on mixed carbon sources

## Supplementary Information

Xin Wang et al.

Supplementary Note 1. Model framework ........ 1
Supplementary Note 1.1 Optimization principles ........ 1
Supplementary Note 1.2 Carbon flux, enzyme cost and substrate quality ........ 1
Supplementary Note 1.3 Biomass components and precursor pools ........ 2
Supplementary Note 1.4 Group A and Group B carbon sources ........ 2
Supplementary Note 1.5 Intermediate nodes ........ 3
Supplementary Note 2. The origin of diauxie ........ 4
Supplementary Note 2.1 Simplest model of diauxie ........ 4
Supplementary Note 2.2 Branch efficiency ........ 4
Supplementary Note 2.3 Decision line ........ 6
Supplementary Note 3. The reason for co-utilization ........ 7
Supplementary Note 4. Pool suppliers in the case of co-utilization ........ 9
Supplementary Note 4.1 The original suppliers of pools in the cases of co-utilization ........ 9
Supplementary Note 4.2 Converged flux ........ 9
Supplementary Note 4.3 Energy production ........ 10
Supplementary Note 4.4 Pool suppliers influenced by the TCA cycle ........ 11
Supplementary Note 4.5 Pool suppliers in practice for optimal growth ........ 13
Supplementary Note 4.6 Note on pathways we have considered ........ 14
Supplementary Note 4.7 Spontaneous decarboxylation of oxaloacetate in solution ........ 14
Supplementary Note 4.8 Enzymes of gluconeogenesis ........ 14
Supplementary Note 4.9 Predictions compared with experiments ........ 14
Supplementary Note 5. Reversible reactions ........ 17
Supplementary Note 5.1 Influence of the reversible reactions ........ 18
Supplementary Note 6. Metabolic regulations ........ 19
Supplementary Note 6.1 Enzyme concentration dependent reaction rate ........ 19
Supplementary Note 6.2 Cooperative effect ........ 19
Supplementary Note 6.3 Enzyme inhibitions by metabolites ........ 20
Supplementary Note 6.4 Enzyme regulations by metabolites that permits any function form ........ 22
Supplementary Note 6.5 Exceptional cases ........ 23
Supplementary Note 7. Summary and discussions on the application scope of our analysis framework ........ 24
Supplementary Figures ........ 26
Supplementary Tables ........ 35
Supplementary References ........ 46

## Supplementary Note 1. Model framework

### Supplementary Note 1.1 Optimization principles

We adopt an optimal protein allocation framework similar to that of previous studies[1-4]: Microbes optimize the efficiency of using enzymes through protein allocation. More specifically, microbes maximize the enzyme utilization efficiency $\varepsilon$:

$$\varepsilon \equiv \frac{J_{\text{tot}}}{\Phi_{\text{tot}}}, \tag{1}$$

where $\Phi_{\text{tot}}$ is the total enzyme cost devoted to reactions and $J_{\text{tot}}$ is the total amount of carbon flux.

### Supplementary Note 1.2 Carbon flux, enzyme cost and substrate quality

Consider a biochemical reaction between substrate $S_i$ (with concentration $[S_i]$) and enzyme $E_i$ (with concentration $[E_i]$), assuming that $S_{i+1}$ is the product:

$$E_i + S_i \underset{d_i}{\overset{a_i}{\rightleftarrows}} E_i \cdot S_i \xrightarrow{k_i^{\text{cat}}} E_i + S_{i+1},$$

where $a_i$, $d_i$ and $k_i^{\text{cat}}$ are chemical reaction parameters. The reaction rate (the carbon flux) $v_i$ follows Michaelis–Menten kinetics[5] (assuming that $[S_i] \gg [E_i \cdot S_i]$):

$$v_i = k_i^{\text{cat}} \cdot [E_i] \cdot \frac{[S_i]}{[S_i] + K_i}, \tag{2}$$

where $K_i \equiv \left(d_i + k_i^{\text{cat}}\right) / a_i$. The concentration of $E_i$ is defined as $[E_i] = \frac{N_{E_i}}{V_{\text{cell}}}$, where $V_{\text{cell}}$ is the cell volume and $N_{E_i}$ is the copy number of enzyme $E_i$ within the cell. The carbon flux of this reaction within the cell is:

$$J_i \equiv V_{\text{cell}} \cdot v_i. \tag{3}$$

Denote the molecular weight (MW) of $E_i$ as $m_{E_i}$. To make the enzyme cost dimensionless, we define $m_0$ the MW unit, and $n_{E_i} \equiv m_{E_i} / m_0$ the cost of an $E_i$ molecule. Then the cost of all

$E_i$ molecules within a cell is

$$\Phi_i \equiv N_{E_i} \cdot n_{E_i} = V_{\text{cell}} \cdot [E_i] \cdot n_{E_i} . \tag{4}$$

The substrate quality of $S_i$ for enzyme $E_i$, $\kappa_i$, defined as the efficiency of using $E_i$, according to Supplementary Equation 1, follows:

$$\kappa_i \equiv \frac{J_i}{\Phi_i} = k_i \cdot \frac{[S_i]}{[S_i] + K_i} , \tag{5}$$

where

$$k_i \equiv k_i^{\text{cat}} / n_{E_i} . \tag{6}$$

Since $k_i$ and $K_i$ are constants, then $\kappa_i$ is a function of $[S_i]$.

**Supplementary Note 1.3 Biomass components and precursor pools**

For microbes, biomass consists of multiple components such as proteins, RNA, DNA, lipids, and glycogen, *etc.* (Fig. 1). Based on the topology of metabolic network, we classify the precursors of biomass components into seven pools. Specifically, each pool is named depending on its entrance point on the metabolic network: a1 (entering from G6P/F6P: precursors of RNA, DNA, Glycogen, Lipoglycans, Murein; His, Trp, Phe, Tyr), a2 (entering from GA3P: precursors of RNA, DNA; Trp, Phe, Tyr), a3 (entering from 3PG: Ser, Gly, Cys), a4 (entering from PEP: Trp, Phe, Tyr), b (entering from pyruvate/Acetyle-CoA: Lipids; Ala, Val, Leu, Ile), c (entering from α-Ketoglutarate: Glu, Gln, Pro, Arg) and d (entering from oxaloacetate: Asp, Asn, Met, Thr, Lys). In microbial growth, these seven pools draw roughly $r_{\text{a1}} = 24\%$, $r_{\text{a2}} = 14\%$, $r_{\text{a3}} = 5\%$, $r_{\text{a4}} = 5\%$, $r_{\text{b}} = 28\%$, $r_{\text{c}} = 12\%$ and $r_{\text{d}} = 12\%$ carbon flux, respectively[5-7]. Note that there are some overlapping components between Pools a1, a2, a3 and a4 owing to joint synthesis of precursors. For convenience, we lump sum Pools a1-a4 as Pool a and use the term precursor pools to denote both amino acid pools and the precursor pools for other components.

**Supplementary Note 1.4 Group A and Group B carbon sources**

Denote carbon sources entering the upper part of the glycolysis Group A and those joining at other parts of the metabolic network Group B (Fig. 1). Specifically, all Group A carbon sources join through G6P/F6P. Glycerol enters from the upper part of glycolysis but not G6P/F6P, thus we classify glycerol as a quasi-Group A carbon source. Group A carbon sources can be co-utilized

with Group B sources, whereas substrates paired from Group A usually display diauxie. In most cases, glycerol follow all the traits of Group A sources, yet from the network topology, glycerol can be co-utilized with another Group A source of low concentration such as glucose and lactose (as recently observed in experiment[8]) under optimal conditions.

**Supplementary Note 1.5 Intermediate nodes**

In a real metabolic network (Fig. 1), there are multiple intermediate nodes in delivering carbon flux from carbon sources to precursor pools. We consider a simple case containing one carbon source *A*1 and one intermediate node, *M* (Supplementary Fig. 1a). For *A*1, denote $E_{A1}$ as the catabolic enzyme (with enzyme cost $\Phi_{A1}$), the substrate concentration is $[A1]$ and the substrate quality is $\kappa_{A1} = k_{A1} \cdot \frac{[A1]}{[A1]+K_{A1}}$, where $k_{A1}$ and $K_{A1}$ are constants (similar to that of Supplementary Equation 5). For node *M*, $E_M$ denotes the catabolic enzyme (with enzyme cost $\Phi_M$), and $S_M$ the substrate (with concentration $[S_M]$ and substrate quality $\kappa_M$), where $\kappa_M = k_M \cdot \frac{[S_M]}{[S_M]+K_M}$. Here, $J_{\text{tot}} = \Phi_{A1} \cdot \kappa_{A1} = \Phi_M \cdot \kappa_M$, while $\Phi_{\text{tot}} = \Phi_{A1} + \Phi_M$. Then, $\Phi_{\text{tot}} = J_{\text{tot}} \left(1/\kappa_{A1} + 1/\kappa_M\right)$, and

$$\varepsilon = \frac{1}{1/\kappa_{A1} + 1/\kappa_M}. \tag{7}$$

$\varepsilon$ is clearly a monotonic function of $\kappa_M$ for a given nutrient condition (thus a given value of $\kappa_{A1}$). Combined with Supplementary Equation 5, it is clear that $\varepsilon$ is maximized when $[S_M]$ is nearly saturated:

$$\frac{[S_M]}{[S_M]+K_M} \approx 1, \tag{8}$$

where $K_M$ is the Michaelis–Menten constant, thus $\kappa_M \approx k_M$ (see Supplementary Equation 5). The real situations could be much more complicated (see Supplementary Notes 5-6 for representative cases), the substrate concentration of the intermediate nodes may be not saturated

owning to other constraints. Strikingly, recent studies[9, 10] reported that at least in *E.coli*, metabolite concentration exceeds $K_M$ for most substrate-enzyme pairs. i.e. $[S_M] > K_M$, which implies $\kappa_M \approx k_M$.

## Supplementary Note 2. The origin of diauxie

### Supplementary Note 2.1 Simplest model of diauxie

In the simplest model for diauxie (Supplementary Fig. 1b), the carbon fluxes from substrates $A1$ and $A2$ infuse separately into the precursor pools. $\kappa_{Ai}$ ($i$=1, 2) is the substrate quality of $Ai$ ($i$=1, 2), while $E_{Ai}$ (with enzyme cost $\Phi_{Ai}$, $i$=1, 2) is the carrier enzyme for $Ai$. Here, $J_{\text{tot}} = \Phi_{A1} \cdot \kappa_{A1} + \Phi_{A2} \cdot \kappa_{A2}$, while $\Phi_{\text{tot}} = \Phi_{A1} + \Phi_{A2}$. Then,

$$\varepsilon = \frac{\Phi_{A1} \cdot \kappa_{A1} + \Phi_{A2} \cdot \kappa_{A2}}{\Phi_{A1} + \Phi_{A2}} = \kappa_{A1} - \frac{\Phi_{A2} \cdot (\kappa_{A1} - \kappa_{A2})}{\Phi_{A1} + \Phi_{A2}} = \kappa_{A2} - \frac{\Phi_{A1} \cdot (\kappa_{A2} - \kappa_{A1})}{\Phi_{A1} + \Phi_{A2}}. \tag{9}$$

If $\kappa_{A1} > \kappa_{A2}$, then $\varepsilon = \kappa_{A1} - \frac{\Phi_{A2} \cdot (\kappa_{A1} - \kappa_{A2})}{\Phi_{A1} + \Phi_{A2}} \le \kappa_{A1}$, with $\Phi_{A2} = 0$ the optimal point; if $\kappa_{A1} < \kappa_{A2}$, then $\varepsilon = \kappa_{A2} - \frac{\Phi_{A1} \cdot (\kappa_{A2} - \kappa_{A1})}{\Phi_{A1} + \Phi_{A2}} \le \kappa_{A2}$, with $\Phi_{A1} = 0$ the optimal point. In either case, cells will only use the preferable carbon source, which corresponds to the case of diauxie.

### Supplementary Note 2.2 Branch efficiency

In real cases, multiple intermediate nodes deliver carbon flux before branches converge to a common node. To take into account the cost of the intermediate enzymes, consider a model depicted in Fig. 2b. Here, carbon source $X$ mixed with carbon source $Y$, where $X$ and $Y$ can come from either Group A or Group B. Supposing that there are $N_X$ intermediate nodes ($m_X^j$, $j = 1 \sim N_X$) specifically for $X$ and $N_Y$ intermediate nodes ($m_Y^j$, $j = 1 \sim N_Y$) specifically for $Y$, with carbon fluxes from $X$ and $Y$ merging at node $M$. $M$ can be different for different combinations of $X$ and $Y$. $\kappa_X^j$, $\kappa_Y^j$ and $\kappa_M$ are the substrate quality of nodes $m_X^j$, $m_Y^j$ and $M$, respectively, and $E_X^j$, $E_Y^j$ and $E_M$ the corresponding carrier

enzymes (with enzyme cost $\Phi_X^j$ , $\Phi_Y^j$ and $\Phi_M$ , respectively). Here, $J_{\text{tot}} = \Phi_X \cdot \kappa_X + \Phi_Y \cdot \kappa_Y$, with $\Phi_X \cdot \kappa_X = \Phi_X^j \cdot \kappa_X^j$ $\left( j = 1 \sim N_X \right)$ and $\Phi_Y \cdot \kappa_Y = \Phi_Y^j \cdot \kappa_Y^j$ $\left( j = 1 \sim N_Y \right)$ , while $\Phi_{\text{tot}} = \Phi_X + \sum_{j=1}^{N_X} \Phi_X^j + \Phi_Y + \sum_{j=1}^{N_Y} \Phi_Y^j$ . Then

$$\varepsilon = \frac{\Phi_X \cdot \kappa_X + \Phi_Y \cdot \kappa_Y}{\Phi_X + \sum_{j=1}^{N_X} \Phi_X^j + \Phi_Y + \sum_{j=1}^{N_Y} \Phi_Y^j} = \frac{\Phi_{X \to M}^{\text{b}} \cdot \varepsilon_{X \to M} + \Phi_{Y \to M}^{\text{b}} \cdot \varepsilon_{Y \to M}}{\Phi_{X \to M}^{\text{b}} + \Phi_{Y \to M}^{\text{b}}}, \tag{10}$$

where $\Phi_{X \to M}^{\text{b}} \equiv \Phi_X + \sum_{j=1}^{N_X} \Phi_X^j$ and $\Phi_{Y \to M}^{\text{b}} \equiv \Phi_Y + \sum_{j=1}^{N_Y} \Phi_Y^j$ , while $\varepsilon_{X \to M}$ and $\varepsilon_{Y \to M}$ are defined as the branch efficiency of $X$ and $Y$ to convergent node $M$ , respectively:

$$\varepsilon_{X \to M} = \frac{1}{1/\kappa_X + \sum_{j}^{N_X} 1/\kappa_X^j}, \varepsilon_{Y \to M} = \frac{1}{1/\kappa_Y + \sum_{j}^{N_Y} 1/\kappa_Y^j}. \tag{11}$$

Compare Supplementary Equation 10 with Supplementary Equation 9, it is clear that the supplier of node $M$ depends on the value of $\varepsilon_{X \to M}$ and $\varepsilon_{Y \to M}$ . For $\varepsilon_{X \to M} > \varepsilon_{Y \to M}$ , with $\Phi_{Y \to M}^{\text{b}} = 0$ the optimal point; For $\varepsilon_{X \to M} < \varepsilon_{Y \to M}$ , with $\Phi_{X \to M}^{\text{b}} = 0$ the optimal point. Only the carbon source with higher branch efficiency is utilized to supply the convergent node $M$ .

In the estimation of branch efficiency, $\kappa_X^j \approx k_X^j$ $\left( j = 1 \sim N_X \right)$ and $\kappa_Y^j \approx k_Y^j$ $\left( j = 1 \sim N_Y \right)$ (see Supplementary Equation 8 and Supplementary Note 1.5). $k_X^j$ and $k_Y^j$ are constants (see Supplementary Equation 6). A special case is that when $\varepsilon_{X \to M} \approx \varepsilon_{Y \to M}$ , both branches are equally efficient. From optimality aspect, $X$ and $Y$ can supply node $M$ with any ratio, while from probability aspect, $X$ and $Y$ should supply node $M$ half and half. Rigorously, the application of branch efficiency in determining optimal supplier relies on that $\varepsilon_{X \to M}$ and $\varepsilon_{Y \to M}$ independent of $\Phi_{X \to M}^{\text{b}}$ , $\Phi_{Y \to M}^{\text{b}}$ (see exceptional cases in Supplementary Note 6.5).

When $X$ and $Y$ both come from Group A, from the topology of metabolic network (Fig. 1 or coarse grained version Fig. 2a), the convergent node $M$ resides upstream to all precursor pools (Supplementary Fig. 1c). For example, for $X$ =glucose and $Y$ =galactose, $M$ = Glucose 6-phosphate (G6P); for the same $X$ but $Y$ =fructose, $M$ = fructose 6-phosphate (F6P). This leads to the result that Group A carbon sources are not utilized simultaneously.

**Supplementary Note 2.3 Decision line**

When we say $A1$ is a better carbon source than $A2$, we mean that at saturated concentrations of $A1$ and $A2$, the branch efficiency $\varepsilon_{A1\to M} > \varepsilon_{A2\to M}$ (Supplementary Fig. 1c). However, Supplementary Equation 5 and Supplementary Equation 11 indicate that branch efficiency depends on nutrient concentration $[Ai]$, via the substrate quality $\kappa_{Ai} = k_{Ai}\cdot\frac{[Ai]}{[Ai]+K_{Ai}}$. $\varepsilon_{Ai}$ is a monotonic function of $[Ai]$. Thus a better sugar at low concentration may not be as preferable as a worse sugar at high concentration. As shown in Supplementary Fig. 1c, assuming that there are $N_{A1}$ intermediate nodes ($m_{A1}^j$, $j=1\sim N_{A1}$) specifically for $A1$ and $N_{A2}$ intermediate nodes ($m_{A2}^j$, $j=1\sim N_{A2}$) specifically for $A2$. $\kappa_{A1}^j \approx k_{A1}^j$ and $\kappa_{A2}^j \approx k_{A2}^j$ (see Supplementary Equation 5 and Supplementary Note 1.5) are the substrate quality of $m_{A1}^j$ and $m_{A2}^j$, respectively. Ideally, the decision line to switch the sugar source and thus to turn on $A2$ carrier genes is at

$$\varepsilon_{A1\to M}([A1]) = \varepsilon_{A2\to M}([A2]). \tag{12}$$

which is

$$1/\kappa_{A1} = 1/\kappa_{A2} + \left(\sum_j^{N_{A2}} 1/k_{A2}^j - \sum_j^{N_{A1}} 1/k_{A1}^j\right), \tag{13}$$

Substituting $\kappa_{Ai} = k_{Ai}\cdot\frac{[Ai]}{[Ai]+K_{Ai}}$, Supplementary Equation 12 is reduced to

$$[A1] = \frac{\delta\cdot[A2]}{\Delta+[A2]}, \tag{14}$$

where $\delta = \frac{K_{A1}}{c_{A1}^{A2} \cdot k_{A1}}$ and $\Delta = \frac{K_{A2}}{c_{A1}^{A2} \cdot k_{A2}}$, and $c_{A1}^{A2}$ is defined as $c_{A1}^{A2} \equiv \frac{1}{\varepsilon_{A2}^{\max}} - \frac{1}{\varepsilon_{A1}^{\max}}$, with $\varepsilon_{Ai}^{\max} = \frac{1}{1/k_{Ai} + \sum_{j}^{N_{Ai}} 1/k_{Ai}^{j}}$ the maximum efficiency of the nutrient *Ai* (at saturating nutrient concentration). $k_{Ai}$ is defined according to Supplementary Equation 6. Since $\varepsilon_{A1}^{\max} > \varepsilon_{A2}^{\max}$, thus $c_{A1}^{A2}$, $\delta$ and $\Delta$ are all positive constants. When $[A2]$ is small ($[A2] \ll \Delta$), the decision line (Supplementary Equation 14) is reduced to $[A1] = \frac{\delta}{\Delta} \cdot [A2]$. That is, the decision line depends only on the ratio of the two nutrients: the cell will sense not the absolute concentration of $[A1]$ and $[A2]$, but their ratio, to make the decision. Ratio sensing was recently observed in the budding yeast *Saccharomyces cerevisiae* cultured in glucose-galactose mixed medium[11]. The measured turning point agrees remarkably well with Supplementary Equation 14 (see Supplementary Fig. 2).

Meanwhile, the mechanism of ratio sensing demands resources. It could well be that the microbe cares only about the most frequently encountered (or the most important) combinations of nutrients and would not invest resources to ratio sense the others.

## Supplementary Note 3. The reason for co-utilization

The topologies of the metabolic network with one carbon source from Group A and the other from Group B are shown in Supplementary Fig. 3a-c. Note a Group B carbon source has the possibility to be co-utilized with another source from Group B. A topology of this type is shown in Supplementary Fig. 3d. These diagrams can be analyzed with the coarse grained model of Supplementary Fig. 1d, which has two precursor pools. In synthesizing biomass, portion $r_1$ of carbon flux comes from Pool 1 and $r_2$ from Pool 2. All intermediate nodes are lump summed into two intermediate nodes: $M$ and $N$, which can convert to each other with the help of respective enzymes: $E'_M$ and $E'_N$. $\kappa_A$, $\kappa_B$, $\kappa_M$, $\kappa_N$, $\kappa'_M$ and $\kappa'_N$ represent substrate

quality, while $\Phi_A$, $\Phi_B$, $\Phi_M$, $\Phi_N$, $\Phi'_M$ and $\Phi'_N$ denote protein cost of carrier enzymes. Here, $\Phi_{\text{tot}} = \Phi_A + \Phi_B + \Phi_M + \Phi_N + \Phi'_M + \Phi'_N$ , $J_{\text{tot}} = \Phi_M \cdot \kappa_M + \Phi_N \cdot \kappa_N$ with constraints: $\Phi_M \cdot \kappa_M + \Phi'_M \cdot \kappa'_M = \Phi_A \cdot \kappa_A + \Phi'_N \cdot \Phi'_N$ , $\Phi_N \cdot \kappa_N + \Phi'_N \cdot \kappa'_N = \Phi_B \cdot \kappa_B + \Phi'_M \cdot \kappa'_M$ and $\frac{\Phi_M \cdot \kappa_M}{\Phi_N \cdot \kappa_N} = \frac{r_1}{r_2}$. To maximize enzyme utilization efficiency $\varepsilon = \frac{J_{\text{tot}}}{\Phi_{\text{tot}}}$, it is equivalent to maximize the enzyme utilization efficiency of every precursor pools (Pool 1 and Pool 2 in Supplementary Fig. 1d), where we can apply branch efficiency analysis (see Supplementary Note 2.2 for details) -- only the carbon source with the highest branch efficiency will be employed to supply the convergent node. At the convergent node *M*: $\varepsilon_{A\to M} = \frac{1}{1/\kappa_A}$, $\varepsilon_{B\to M} = \frac{1}{1/\kappa_B + 1/\kappa'_N}$.

The nutrient supplier of Pool 1 is then determined by

$$\text{Pool 1 is supplied by } \begin{cases} A, \text{if } \varepsilon_{A\to M} > \varepsilon_{B\to M} \\ B, \text{if } \varepsilon_{A\to M} < \varepsilon_{B\to M} \end{cases}. \tag{15}$$

At the intersection node *N*: $\varepsilon_{A\to N} = \frac{1}{1/\kappa_A + 1/\kappa'_M}$, $\varepsilon_{B\to N} = \frac{1}{1/\kappa_B}$. The provider of Pool 2 is then determined according to

$$\text{Pool 2 is supplied by } \begin{cases} A, \text{if } \varepsilon_{A\to N} > \varepsilon_{B\to N} \\ B, \text{if } \varepsilon_{A\to N} < \varepsilon_{B\to N} \end{cases}. \tag{16}$$

If

$$1/\kappa_B - 1/\kappa'_M < 1/\kappa_A < 1/\kappa_B + 1/\kappa'_N , \tag{17}$$

then *A* supplies Pool 1 and *B* provides Pool 2, both substrates are co-utilized. In this case, the enzyme utilization efficiency $\varepsilon$ of the mixed medium *A*+*B* is (see Supplementary Equation 1)

$$\varepsilon_{A+B} = \frac{1}{r_1/\kappa_A + r_1/\kappa_M + r_2/\kappa_B + r_2/\kappa_N}, \tag{18}$$

while the enzyme utilization efficiency $\varepsilon$ of a single substrate medium like *A*1 is

$$\varepsilon_A = \frac{1}{1/\kappa_A + r_1/\kappa_M + r_2/\kappa_N + r_2/\kappa'_M}. \tag{19}$$

In the real case, $1/\kappa'_M$ delegates the summation of intermediate node terms between *M* and *N*, which is often quite large (Supplementary Table 2). As a result, $\varepsilon_{A+B}$ can be significantly greater than $\varepsilon_A$, meaning that co-utilization is commonly the optimal choice when a Group A carbon source mixed with a Group B carbon source.

## Supplementary Note 4. Pool suppliers in the case of co-utilization

### Supplementary Note 4.1 The original suppliers of pools in the cases of co-utilization

The original pool suppliers are determined by the branch efficiencies. We collected the available biochemical parameters from published data (Supplementary Table 1) to estimate the branch efficiencies from carbon sources to precursor pools in *E. coli* (Supplementary Table 2).

In our estimation, for convenience, we only consider carbon sources (e.g. glucose, lactose, pyruvate etc.) with saturated concentrations. For intermediate metabolites, since $[S_M] > K_M$ is valid for most substrate-enzyme pairs in *E.coli*[9, 10], we estimate the substrate quality $\kappa_i \approx k_i = k_i^{\text{cat}} / n_{E_i}$ (see Supplementary Equation 6 and Supplementary Note 1.5). $k_i^{\text{cat}}$ is the turnover number of the enzyme $E_i$, $n_{E_i} = m_{E_i} / m_0$ (see Supplementary Note 1.2) with $m_{E_i}$ the MW of $E_i$ and $m_0$ a MW unit. For convenience, we set $m_0 = 100$ kDa and then can obtain branch efficiency from carbon sources to the counterpart of node *M* and *N* (F6P, GA3P, 3PG, PEP, pyruvate or oxaloacetate) of Pools a1-a4, b and d (Supplementary Table 2). Using branch efficiency analysis (see Supplementary Note 2.2), we get the original suppliers of each pool in different combinations of co-utilization (Supplementary Table 3). Take the case of glucose-pyruvate co-utilization for example (Supplementary Table 3 and Supplementary Fig. 4a), the original supplier of Pools a1-a4 and d is glucose, while the original supplier of Pool b is pyruvate. Due to converged flux, the original supplier of Pool c is not set by the branch efficiency from carbon sources to Pool c, but rather both the suppliers of Pool b (pyruvate) and Pool d (glucose) (see Supplementary Note 4.2 for details). It is worth noting that owing to energy production in the TCA cycle, the pool suppliers in practice can be different from its original supplier (see Supplementary Notes 4.3-4.5 for details).

### Supplementary Note 4.2 Converged flux

(a) Pool c

Pool c is supplied by joint fluxes from pyruvate and oxaloacetate (Supplementary Fig. 4b, pyruvate→ Acetyl-CoA, Acetyl-CoA + oxaloacetate → citrate), which are *M* and *N* node counterpart of Pools b and d, respectively, thus Pool c suppliers are both that of Pools b and d, with 2/5 of carbons supplied from Pools b and 3/5 of carbons supplied from Pools d.

(b) Isoleucine

Isoleucine is synthesized through the following reactions[5]:

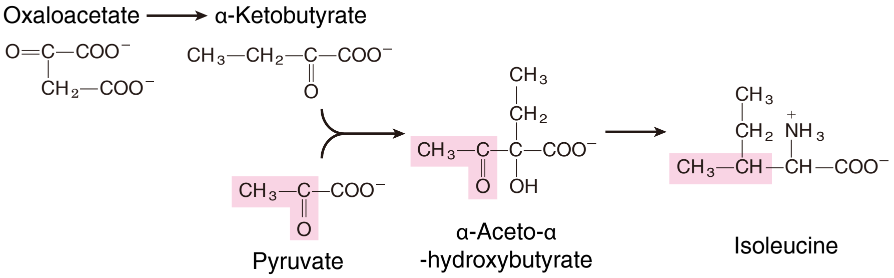


,

with 2/5 of carbons supplied from Pools b and 3/5 of carbons supplied from Pools d.

(c) Tryptophan, Tyrosine & Phenylalanine

Tryptophan, tyrosine and phenylalanine are synthesized through the following reactions[5]:

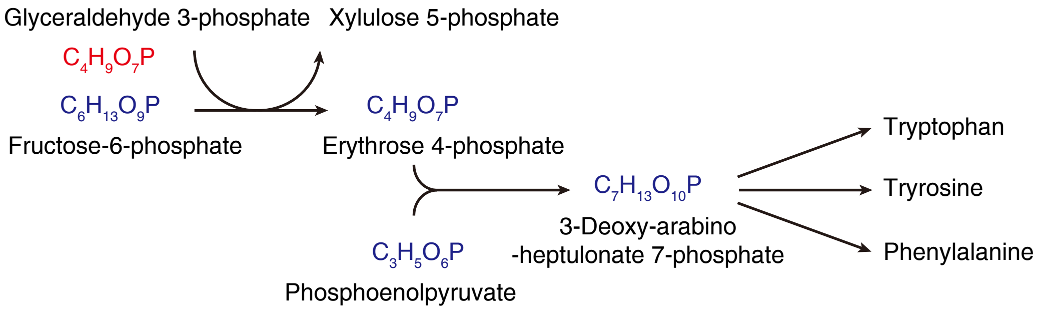


,

with roughly 3/7 of carbons supplied from phosphoenolpyruvate (PEP) and 4/7 of carbons supplied from fructose-6-phosphate (F-6-P).

**Supplementary Note 4.3 Energy production**

In microbial growth, a considerable amount of carbon sources needs to be allocated for energy production. Adenosine triphosphate (ATP), the molecular unit of energy currency, facilitates intracellular energy transfer. Taking the growth of *E. coli* in glucose as an example, it is estimated that 1-2×$10^9$ glucose molecules (7×$10^9$ carbon atoms/cell, BNID 103010) are required to build the biomass of a new cell[12], whereas the amount of carbon sources required for energy production varies depending on the respiration type. For typical aerobic respiration, the energetic

requirement for a new cell is estimated to be 3–6$\times 10^8$ glucose molecules (1.2-1.7$\times 10^{10}$ ATP/cell, BNID 101981, 101983) on top of the molecules needed for the biomass[12-14]. For anaerobic respiration, 3–6$\times 10^9$ glucose molecules[12] are estimated to be required for energy production of a new cell. For aerobic microbial growth in mixed carbon sources with saturated carbon source concentrations, the ratio of energy/biomass allocation is estimated to be 20%-50%[12-14], and we denote this ratio as $r_{\mathrm{E/M}}$.

**Supplementary Note 4.4 Pool suppliers influenced by the TCA cycle**

In TCA cycle, oxaloacetate goes back to itself if reactions flow through the whole cycle (with production of $CO_2$ and ATPs). Consider a newly synthesized oxaloacetate molecule coming from the original supplier of Pool d before the TCA cycle, as depicted in Supplementary Fig. 4b, after 1 round of TCA cycle, half of the carbon atoms in oxaloacetate are replaced by those coming from pyruvate (supplier of Pool b), which means that in practice the supplier of Pool d would be a combination of the original supplier of Pool d and the supplier of Pool b. For clarity, we denote the original supplier of Pool d as $d_{\mathrm{S}}^{\mathrm{O}}$, the original supplier of Pool b as $b_{\mathrm{S}}^{\mathrm{O}}$ and the supplier of Pool d in practice as $d_{\mathrm{S}}^{\mathrm{I}}$. Clearly, $d_{\mathrm{S}}^{\mathrm{I}}$ is a combination of $b_{\mathrm{S}}^{\mathrm{O}}$ and $d_{\mathrm{S}}^{\mathrm{O}}$, and we assume that $\xi$ fraction of carbon atoms in $d_{\mathrm{S}}^{\mathrm{I}}$ coming from $d_{\mathrm{S}}^{\mathrm{O}}$, with the rest $1-\xi$ carbon atoms coming from $b_{\mathrm{S}}^{\mathrm{O}}$.

To quantify the influence of TCA cycle on $\xi$, we consider microbes of exponential growth at certain growth rate, with the TCA cycle depicted in Supplementary Fig. 4c. Since biomass production and energy production are continuous, we assume the total stoichiometry of carbon flux at oxaloacetate is 1 per unit time $\tau$. $r_{\mathrm{d}}'$ stoichiometry of the flux flows to Pool d, while $1-r_{\mathrm{d}}'$ stoichiometry of flux flows to citrate. Owing to the stoichiometry of reactions (pyruvate→ Acetyl-CoA and Acetyl-CoA + oxaloacetate → citrate), $1-r_{\mathrm{d}}'$ stoichiometry of carbon flux would come from citrate per $\tau$. Meanwhile, $r_{\mathrm{c}}'$ stoichiometry of the flux flows to Pool c, with the rest $1-r_{\mathrm{c}}'-r_{\mathrm{d}}'$ stoichiometry of carbon flux flows back to oxaloacetate. To keep sustainable

microbial growth, $r_c' + r_d'$ stoichiometry of carbon flux per $\tau$ would join oxaloacetate from $d_S^O$ so that the stoichiometry of flux at oxaloacetate can be 1 (our assumption from beginning). For microbial growth at fixed growth rate, the system can be treated as non-equilibrium steady state[15], then $\xi$ is under the constraint of the following equation:

$$\xi = \left(r_c' + r_d'\right) + \left(1 - r_c' - r_d'\right)\frac{\xi}{2}, \tag{20}$$

which means that the value of $\xi$ should be the same after 1 round of TCA cycle. The 1/2 in Supplementary Equation 20 derives from the fact that half of the carbon atoms in oxaloacetate have been replaced by that of pyruvate through a TCA cycle. We can solve Supplementary Equation 20 and get:

$$\xi = 2\frac{r_c' + r_d'}{1 + r_c' + r_d'}. \tag{21}$$

In fact, we can estimate the values of $r_c'$ and $r_d'$ using $r_c$, $r_d$ and $r_{E/M}$. Note that $r_c'$ and $r_d'$ denote the stoichiometry ratios of carbon flux. At the point of oxaloacetate before a TCA cycle, 1 stoichiometry flux of oxaloacetate (with 4 carbon atoms per molecule) corresponds to 4 fluxes of carbon atoms. Then, $r_d'$ stoichiometry of oxaloacetate means $4r_d'$ of carbon atoms flow to Pool d. Because of the stoichiometry of reaction (Acetyl-CoA + oxaloacetate →citrate), another $2\left(1 - r_d'\right)$ of carbon atoms join from Acetyl-CoA (Supplementary Fig. 4b-c), this results in $6\left(1 - r_d'\right)$ of carbon atoms at citrate. Later on, owing to carbon leakage in the forms of carbon dioxide ($CO_2$), $5r_c'$ of carbon atoms flows to Pool c through α-ketoglutarate (with 5 carbon atoms per molecule), with finally $4\left(1 - r_c' - r_d'\right)$ of carbon atoms flowing back to oxaloacetate. Clearly, the carbon atoms flowing to Pools c & d are $5r_c'$ and $4r_d'$ per $\tau$, respectively. Meanwhile, the carbon flux allocated for energy production can be estimated from the production of $CO_2$. As shown in Supplementary Fig. 4b-c, $CO_2$ is generated of $1 - r_d'$ in reaction pyruvate→ Acetyl-CoA; $1 - r_d'$ in reaction of isocitrate→α-ketoglutarate, and $1 - r_c' - r_d'$ in reaction

α-ketoglutarate→Succinyl-CoA, with $3-r_c'-3r_d'$ per $\tau$ in total. We roughly estimate this to be the energy allocation for generating ATPs. Then,

$$\frac{r_{E/M}}{3-r_c'-3r_d'}=\frac{r_c}{5r_c'}=\frac{r_d}{4r_d'}, \tag{22}$$

thus

$$\begin{cases} r_c'=\dfrac{3r_c}{5r_{E/M}+r_c+3.75r_d} \\ r_d'=\dfrac{3.75r_d}{5r_{E/M}+r_c+3.75r_d} \end{cases}. \tag{23}$$

Substitute Supplementary Equation 23 into Supplementary Equation 21, we get:

$$\xi=\frac{6r_c+7.5r_d}{5r_{E/M}+4r_c+7.5r_d}. \tag{24}$$

Since $r_c \approx 0.12$, $r_d \approx 0.12$, $0.2 \le r_{E/M} \le 0.5$ (Supplementary Notes 1.3 & 4.3), the value of $\xi$ can be estimated from Supplementary Equation 24:

$$0.42 \le \xi \le 0.68. \tag{25}$$

Combined with the experiment data we obtained, we roughly estimate $\xi$ to be 0.55 for all the aerobic growth with saturated carbon source concentrations.

**Supplementary Note 4.5 Pool suppliers in practice for optimal growth**

To summarize, for optimal growth of *E.coli*, $\xi$ (≈55%) of the carbons in Pool d (Pool d supplier in practice $d_S^I$) come from its original supplier $d_S^O$ (determined by its branch efficiency), $1-\xi$ (≈45%) of the carbons in Pool d come from the supplier of Pool B $b_S^O$. Meanwhile, Pool c is supplied by joining fluxes from pyruvate (2/5) and oxaloacetate (3/5). Consequently, $0.6\xi$ (≈33%) of the carbons in Pool c come from $d_S^O$ while $1-0.6\xi$ (≈67%) of the carbons in Pool c come from $b_S^O$. Model predictions of the pool suppliers for some combinations of mixtures are listed in Supplementary Table 4.

**Supplementary Note 4.6 Note on pathways we have considered**

Besides the metabolite pathways listed in Fig. 1 (with enzyme parameters shown in Supplementary Table 1), we have considered other pathways such as Entner-Doudoroff pathway and Glyoxylate cycle[5], yet the inclusion of these pathways do no change the model predictions of carbon source suppliers for the combinations of mixtures we have considered.

**Supplementary Note 4.7 Spontaneous decarboxylation of oxaloacetate in solution**

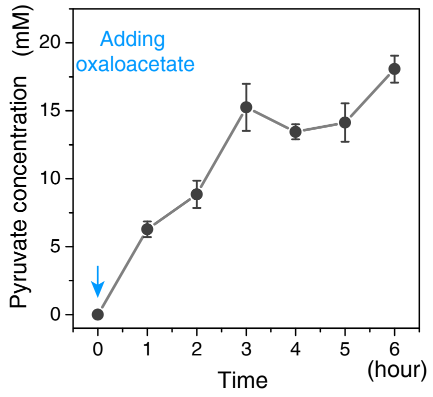


Figure. Oxaloacetate spontaneously decarboxylates into pyruvate in solution. Oxaloacetate powders were added into solution at time 0. Each dot is an average over three independent experiments, error bars represent standard deviations. Source data are provided as a Source Data file.

Oxaloacetate can spontaneously decompose to pyruvate and $CO_2$ when added into solution[16]. We confirmed this result with our experiment (shown above) and found that the quick decarboxylation of oxaloacetate would result in high concentration of pyruvate. This essentially changes the types of carbon sources in the culturing medium. As a result, we do not predict combinations including oxaloacetate.

**Supplementary Note 4.8 Enzymes of gluconeogenesis**

For optimal growth, there should be no gluconeogenesis enzymes when there is carbon flux of glycolysis. In reality, microbes need to dealing with the frequently varying environments. Empirically, it was found in *E.coli* that microbes reserve a portion of gluconeogenesis enzymes when using Group A carbon sources, with the enzymes expression level anti-correlated with the carbon fluxes down through the glycolysis[17]. This may enable microbes to balance growth and prepare for potential changing environments[17].

**Supplementary Note 4.9 Predictions compared with experiments**

The comparison of experimental results with predictions of the in-practice pool suppliers (Supplementary Table 4) is shown in Figs. 3-4 & Supplementary Figs. 5-6. For all these combinations, the experimental results quantitatively agree with the model predictions. The slight differences between model predictions and experimental results in Pool a (combining Pool a1-a4) supply might be due to the fact that microbes reserve a portion of gluconeogenesis enzymes to prepare for potential changing environment[17].

In our model predictions, all parameters were collected or estimated from published literatures (Supplementary Table 1). For some transporters with no direct experimental data, we estimated the order of magnitude for their parameters[12] (labeled as "Estimated" in Supplementary Table 1). Admittedly the reported turnover numbers (or $k_{cat}$ values) of enzymes are likely to be associated with errors (e.g. measurement errors and *in vitro* versus *in vivo* errors), and the estimated parameters for transporters probably involve even large errors. However, it is still rather nontrivial to observe the consistency between model predictions (Supplementary Table 4) and experimental data (Figs. 3-4 & Supplementary Figs. 5-6) as we explain it below.

In Supplementary Tables 6 and 7, we list all possible patterns of carbon source distribution for both of the original pool suppliers (Supplementary Table 6) and of the in-practice pool suppliers (Supplementary Table 7) of *E. coli*, respectively, where we have considered all possibilities for the choice of the biochemical parameters ($k_{cat}$ can be any positive values for all enzymes involved). For each combination of carbon sources, there are only a handful of possible patterns (Supplementary Tables 6 and 7), since there can only be one-zero or half-half (when the branch efficiencies of two substrates are roughly the same for a precursor pool) supply pattern for the original supplier of a precursor pool and the branch efficiency of every carbon source decreases when incorporating more intermediate nodes. Evidently, all possible patterns are very distinct from each other (quantized), and actually there is no way to freely fit even a single pattern in any mixture of two carbon sources by tuning parameters. To illustrate this point, we focus on possible patterns of the in-practice pool suppliers (Supplementary Table 7) that are directly comparable to experimental results.

In the case that a Group A carbon source mixed with B1 (pyruvate) (A+B1 in Supplementary Table 7), there are two possible patterns of diauxie (DX Nos. 1-2) and 13 possible patterns of co-utilization (CoU Nos. 1-13). However, only three patterns (CoU No. 1, CoU No. 8 and CoU No. 9 in A+B1) are qualitatively similar to experimental results (A+B1); other patterns are very different in at least one of the pools (Pool a-d). Among the three similar patterns, CoU No. 1 (in

A+B1) is the pattern predicted for Glucose/Lactose + B1 (Supplementary Table 4); CoU No. 9 (in A+B1) is the pattern predicted for Fructose/Glycerol + B1 (Supplementary Table 4); CoU No. 8 (in A+B1) is very similar to CoU No. 9 (in A+B1), yet one necessity for CoU No. 8 is that pyruvate is more efficient to supply oxaloacetate (entry point of Pool d) via malate than the other route via PEP (entry point of Pool a4), which requires at least 10-fold increase in the $k_{cat}$ value (in the reaction: Pyruvate →Malate) from the nominal value based on published literatures (Supplementary Table 1). Obviously, there is no free space for parameter fitting, and this situation also holds for most mixed combinations.

In the case that a Group A carbon source mixed with B2 (Succinate/ Malate/ Fumarate) (A+B2 in Supplementary Table 7), there are two possible patterns of diauxie (DX Nos. 1-2) and 17 possible patterns of co-utilization (CoU Nos. 1-17). For Glucose/Lactose + B2, only two possible patterns are qualitatively similar with the experiment: CoU No. 1 and CoU No. 8 (in A+B2). Here CoU No. 1 (in A+B2) is the predicted pattern; CoU No. 8 (in A+B2) is actually possible when tuning the $k_{cat}$ values of B2 transporters (requires a reduction of roughly forty percent, in Supplementary Table 1). Thus, in this case, tuning parameters can facilitate a choice between CoU No. 1 and CoU No. 8 (in A+B2), yet there is no other choice for a qualitatively similar pattern. Quantitatively, CoU No. 8 (in A+B2) is distinguishable from CoU No. 1 (in A+B2) and different from the experimental pattern. For Fructose/Glycerol + B2, there are four patterns qualitatively similar with experiments: CoU No. 7, CoU No. 9, CoU No. 11 and CoU No. 13 (in A+B2). Here CoU No. 9 (in A+B2) is the predicted pattern. CoU No. 13 (in A+B2) is very similar to CoU No. 9 (in A+B2), yet CoU No. 13 requires more than 5-fold increase in the $k_{cat}$ value (in the reaction: oxaloacetate →PEP) from the nominal value based on published literatures (Supplementary Table 1). CoU No. 7 and CoU No. 11 (in A+B2) are quantitatively distinguishable from CoU No. 9 (in A+B2). Based on current parameters in Supplementary Table 1, A owns higher branch efficiency at pyruvate (entry point of Pool b) than that of oxaloacetate (entry point of Pool d), then both CoU No. 7 and CoU No. 11 (in A+B2) would require that B2 owns a higher branch efficiency at pyruvate than that of oxaloacetate, which means a minimum 30-fold increase in the $k_{cat}$ value (in the reaction: Malate →Pyruvate) from the nominal value based on published literatures (Supplementary Table 1). Meanwhile, to make A with a higher branch efficiency at oxaloacetate than that of pyruvate would require at least 6-fold increase in the $k_{cat}$ value (in the reaction: PEP →Oxaloacetate) from the nominal value based on published literatures (Supplementary Table 1). Furthermore, CoU No. 11 (in A+B2) additionally requires that the branch efficiencies from A and B2 equal to each other both at pyruvate and oxaloacetate via two independent equalities.

In the case that B1 (pyruvate) mixed with B2 (Succinate/ Malate/ Fumarate) (B1+B2 in Supplementary Table 7), two patterns are qualitatively similar with experiments: CoU No. 1 and CoU No. 3 (in B1+B2). Here CoU No. 1 (in B1+B2) is the predicted pattern; CoU No. 3 (in B1+B2) is also possible when tuning the $k_{\mathrm{cat}}$ values of B2 transporters (requires a reduction of roughly forty percent, in Supplementary Table 1), and quantitatively it is distinguishable from CoU No. 1 (in B1+B2) and different from the experimental pattern.

Finally, in the case that Succinate mixed with Malate (Succinate + Malate in Supplementary Table 7), CoU No. 1 (Succinate + Malate) is the only co-utilized pattern and also the predicted pattern, which quantitatively agree with experimental results.

Summarily, for any mixture, only a very limited number (no more than 4) of possible patterns can qualitatively agree with experiments. While among these qualitatively similar patterns, if the associated errors of the $k_{\mathrm{cat}}$ (in Supplementary Table 1) are less than 5-fold, tuning parameters can only facilitate two choices: one between CoU No. 1 and CoU No. 8 in A+B2 and the other between CoU No. 1and CoU No. 3 in B1+B2. Thus, tuning parameters cannot freely fit pool supply patterns. Consequently, the consistency between model predictions (Supplementary Table 4 or blue text lines in Supplementary Table 7) and experimental data in all combinations of mixtures considered demonstrates the usefulness of our theory.

Furthermore, besides the *E. coli* data from our experiments, published experiment data[18] in *Methylobacterium extorquens AM1* is qualitatively highly consistent with our model. Their carbon supply pattern (Figure 3 in the reference article[18]) is very similar to that of Supplementary Fig. 4a.

## Supplementary Note 5. Reversible reactions

Reversible reactions are common in metabolic network (Fig. 1). To analyze the influence of this factor, we consider the scheme that

$$S_i \xleftrightarrow{E_i} S_{i+1},$$

where $E_i$ is the enzyme catalyzing the reversible reaction between substrate $S_i$ and $S_{i+1}$. We can approximate the details of the reaction as follows:

$$\begin{cases} S_i + E_i \underset{d_i^{\rightarrow}}{\overset{a_i^{\rightarrow}}{\rightleftarrows}} E_i \cdot S_i \xrightarrow{k_i^{\rightarrow}} E_i + S_{i+1} \\ S_{i+1} + E_i \underset{d_i^{\leftarrow}}{\overset{a_i^{\leftarrow}}{\rightleftarrows}} E_i \cdot S_{i+1} \xrightarrow{k_i^{\leftarrow}} E_i + S_i \end{cases},$$

Here $a_i^{\rightarrow}$, $a_i^{\leftarrow}$, $d_i^{\rightarrow}$, $d_i^{\leftarrow}$, $k_i^{\rightarrow}$ and $k_i^{\leftarrow}$ are the chemical reaction parameters, the superscripts $\rightarrow$ and $\leftarrow$ stand for forward and reverse reactions, respectively. The net reaction rate $v_i$ from $S_i$ to $S_{i+1}$ follows the Michaelis-Menten equation of the reversible form[19, 20]:

$$v_i = \frac{k_i^{\rightarrow}[S_i]/K_i^{\rightarrow} - k_i^{\leftarrow}[S_{i+1}]/K_i^{\leftarrow}}{1+[S_i]/K_i^{\rightarrow} + [S_{i+1}]/K_i^{\leftarrow}}[E_i], \tag{26}$$

where $K_i^{\rightarrow} \equiv \left(d_i^{\rightarrow} + k_i^{\rightarrow}\right)/a_i^{\rightarrow}$, and $K_i^{\leftarrow} \equiv \left(d_i^{\leftarrow} + k_i^{\leftarrow}\right)/a_i^{\leftarrow}$. Then, the substrate quality of $S_i$:

$\kappa_i = \frac{k_i^{\rightarrow}[S_i]/K_i^{\rightarrow} - k_i^{\leftarrow}[S_{i+1}]/K_i^{\leftarrow}}{1+[S_i]/K_i^{\rightarrow} + [S_{i+1}]/K_i^{\leftarrow}} < k_i^{\rightarrow}$ . Note that when $[S_i]/K_i^{\rightarrow} > [S_{i+1}]/K_i^{\leftarrow} > 1$ (rigorously $[S_i]/K_i^{\rightarrow} \gg [S_{i+1}]/K_i^{\leftarrow} \gg 1$), $\varepsilon$ is maximized and $\kappa_i \approx k_i^{\rightarrow}$, which means that at optimal conditions analysis applies to irreversible cases is valid for reversible reactions.

One prediction of the reversible reaction analysis is that metabolites at the upper part of glycolysis (e.g. G6P, fructose 1,6-bisphosphatase (FBP)) owns a much higher concentration when bacteria cultured in Group A carbon sources (e.g. glucose) compared to that bacteria cultured in carbon sources entering from lower parts of glycolysis or TCA cycle. Recent studies[17] found that G6P and FBP have a much higher concentration when bacteria cultured with glucose than that shifting into acetate (entering from the bottom of glycolysis), which agree well with our reversible reaction analysis.

**Supplementary Note 5.1 Influence of the reversible reactions**

Note that optimal conditions operate at a non-equilibrium steady state that metabolic flux coming from external carbon sources working at maximum rate. Were the carbon flux drops for a while (e.g. bacteria take a short rest when consuming sugars), the reversible reaction between $S_i$ and $S_{i+1}$ would be quickly in equilibrium. This ideally can make the pools with reversible interconverting entry metabolites (counterpart of node *M* and *N*) be at similar carbon supply percentages. For the synthesis of biomass, Pools a2-a4 can be affected by this effect which makes

the in-practice suppliers of Pools a2-a4 very similar.

## Supplementary Note 6. Metabolic regulations

### Supplementary Note 6.1 Enzyme concentration dependent reaction rate

Consider again enzyme reaction:

$$S_i + E_i \underset{d_i}{\overset{a_i}{\rightleftarrows}} E_i \cdot S_i \xrightarrow{k_i^{\text{cat}}} E_i + S_{i+1}.$$

The Michaelis-Menten enzyme kinetics $v_i = k_i^{\text{cat}} \frac{[S_i]}{[S_i]+K_i}[E_i]$ actually relies on the assumption that $[S_i] \gg [E_i \cdot S_i]$ [5], yet the precise form of the reaction rate is enzyme concentration dependent[21, 22]:

$$v_i = k_i^{\text{cat}} \frac{([S_i]+[E_i]+K_i)}{2}\left(1-\sqrt{1-\frac{4[S_i][E_i]}{([S_i]+[E_i]+K_i)^2}}\right) \approx k_i^{\text{cat}} \frac{[S_i][E_i]}{[E_i]+[S_i]+K_i}. \quad (27)$$

Here we apply approximation $\sqrt{1-x} \approx 1-x/2$ where $x \equiv \frac{4[S_i][E_i]}{([S_i]+[E_i]+K_i)^2} < 1$. When $\varepsilon$ is maximized, $[S_i] \gg K_i, [E_i]$, and $\kappa_i \approx k_i$. Thus the analysis framework in the Supplementary Note 1 and the branch efficiency analysis (Supplementary Note 2.2) are valid for this case.

### Supplementary Note 6.2 Cooperative effect

Chemical reactions are subject to multiple regulations in the metabolite network. Allosteric enzymes, for instance, can introduce Hill coefficients in the kinetics of chemical reactions[5]. Consider the following scheme:

$$nS_i + E_i \underset{d_i}{\overset{a_i}{\rightleftarrows}} E_i \cdot nS_i \xrightarrow{k_i^{\text{cat}}} E_i + nS_{i+1}.$$

The reaction rate $v_i$ follows (assuming that $[S_i] \gg [E_i \cdot nS_i]$):

$$v_i = k_i^{\text{cat}} \frac{[S_i]^n}{[S_i]^n + K_i}[E_i], \quad (28)$$

where $K_i \equiv (d_i + k_i)/a_i$. Then, the substrate quality of $S_i$: $\kappa_i = k_i \frac{[S_i]^n}{[S_i]^n + K_i}$ (see Supplementary Equation 6). When $\varepsilon$ is maximized, $[S_i] > K_M{}^{-n}$ and $\kappa_i \approx k_i^{\rightarrow}$, which means that cooperative effect applies to analysis framework in Supplementary Note 1 and the branch efficiency analysis (Supplementary Note 2.2).

**Supplementary Note 6.3 Enzyme inhibitions by metabolites**

There are two general classes of enzyme inhibitors: reversible inhibitors and irreversible inhibitors.

Irreversible inhibitors are mostly small molecules. For metabolites, in principle, are allowed to take this role by inhibiting metabolic reactions of other branches (e.g. catabolic repression in the case of diauxie); however, as we show it below, they are quite unlikely to take this role in their own metabolic branches. Supposing that $S_j$ is a downstream metabolite of reaction $S_i \rightarrow S_{i+1}$ (with $E_i$ is the catalyzing enzyme), while functioning as an irreversible inhibitor:

$$\cdots \rightarrow S_i \xrightarrow{E_i} S_{i+1} \rightarrow \cdots \rightarrow S_j \rightarrow ,$$

where $S_j$ inhibit $E_i$ irreversibly with reaction:

$$S_j + E_i \rightarrow S_j \cdot E_i .$$

Here the enzyme within $S_j \cdot E_i$ is inactive. Note that $S_j$ is accumulated as long as there is active form of $E_i$, eventually $[S_j] \gg [E_i]$. This mechanism would shut down the metabolic flow through this branch and thus is unlikely to exist.

Reversible inhibitions, generically, have three types[5]: (a) Competitive inhibition

$$\begin{cases} E_i + S_i \underset{d_i}{\overset{a_i}{\rightleftharpoons}} E_i \cdot S_i \xrightarrow{k_i^{\text{cat}}} E_i + S_{i+1} \\ E_i + I \underset{d_i^I}{\overset{a_i^I}{\rightleftharpoons}} E_i \cdot I \end{cases};$$

where $I$ denotes the enzyme inhibitor; (b) Uncompetitive inhibition

$$\begin{cases} E_i + S_i \underset{d_i}{\overset{a_i}{\rightleftarrows}} E_i \cdot S_i \xrightarrow{k_i^{\text{cat}}} E_i + S_{i+1} \\ E_i \cdot S_i + I \underset{d_i^{I'}}{\overset{a_i^{I'}}{\rightleftarrows}} E_i \cdot S_i \cdot I \end{cases};$$

and (c) Mixed inhibition

$$\begin{cases} E_i + S_i \underset{d_i}{\overset{a_i}{\rightleftarrows}} E_i \cdot S_i \xrightarrow{k_i^{\text{cat}}} E_i + S_{i+1} \\ E_i + I \underset{d_i^{I}}{\overset{a_i^{I}}{\rightleftarrows}} E_i \cdot I \\ E_i \cdot I + S_i \underset{d_i}{\overset{a_i}{\rightleftarrows}} E_i \cdot S_i \cdot I \\ E_i \cdot S_i + I \underset{d_i^{I'}}{\overset{a_i^{I'}}{\rightleftarrows}} E_i \cdot S_i \cdot I \end{cases}.$$

Here, competitive inhibition is common in metabolic network (e.g. ATP regulates phosphofructokinase-1 in glycolysis) while the two other types have not been observed for enzymes with a single substrates[5]. For competitive inhibition, the Michaelis-Menten kinetics is reshaped into[5]

$$v_i = k_i^{\text{cat}} \frac{[S_i]}{[S_i] + \gamma K_i} [E_i], \tag{29}$$

where $K_i \equiv \left(d_i + k_i^{\text{cat}}\right) / a_i$, $\gamma \equiv 1 + \frac{[I]}{K_I}$ and $K_I \equiv d_i^I / a_i^I$. $K_I$ should be not very small since the inhibition is reversible. Then, the substrate quality of $S_i$: $\kappa_i = k_i \frac{[S_i]}{[S_i] + \gamma K_i}$ (see Supplementary Equation 6). Supposing that metabolite $S_j$ is the inhibitor $I$, when $\varepsilon$ is maximized, $[S_i] \gg \gamma K_i = K_i + \frac{K_i}{K_I}[S_j]$ while $[S_j] \gg K_j$, then $\kappa_i \approx k_i$ and $\kappa_j \approx k_j$, which means that analysis framework in Supplementary Note 1 and the branch efficiency analysis (Supplementary Note 2.2) are still applicable.

For uncompetitive inhibition, the Michaelis-Menten kinetics becomes

$$v_i = k_i^{\text{cat}} \frac{[S_i]}{\gamma'[S_i] + K_i} [E_i], \tag{30}$$

where $\gamma' \equiv 1+\frac{[I]}{K_I'}$, $K_I' \equiv d_i^{I'} / a_i^{I'}$. In the case of mixed inhibition, the rate equation is

$$v_i = k_i^{\text{cat}} \frac{[S_i]}{\gamma'[S_i]+\gamma K_i}[E_i]. \tag{31}$$

In these two cases (reversible inhibition type b &c), however, $\kappa_i$ is clearly dependent on the concentration of $I$. We discuss more general regulations below including these two cases.

**Supplementary Note 6.4 Enzyme regulations by metabolites that permits any function form**

Here we assume that reaction rates $v_i$ depends linearly on $[E_i]$, all forms of metabolic regulations are permitted that $\kappa_i$ (the substrate quality of $S_i$) can be influenced by any metabolites: i.e. $\kappa_i = \kappa_i([\mathbf{S}])$, where $[\mathbf{S}] = ([S_1],[S_2],...,[S_N])$. This case has been studied with the optimal condition corresponds to elementary flux mode[2, 23, 24]. For our purpose here, when $\varepsilon$ is maximized, the approximation of $\kappa_i \approx k_i$ is no longer ensured. However, every metabolite owns an optimal case specific concentration: $[\mathbf{S}] = [\mathbf{S}^0]$ (i.e. $([S_1],[S_2],...,[S_N]) = ([S_1^0],[S_2^0],...,[S_N^0])$. $[\mathbf{S}^0]$ is culturing medium dependent, yet unique for a given medium with a given nutrient concentration, and thus $\kappa_i = \kappa_i([\mathbf{S}^0])$. In this case, we can regard $\kappa_i([\mathbf{S}^0])$ as a medium (with a given nutrient concentration) specific parameter at optimal conditions, branch efficiency analysis qualitatively applies (e.g In Fig.2b and Supplementary Note 2.2, there are fixed value for $\varepsilon_{X\to M}$ and $\varepsilon_{Y\to M}$, yet unable to obtain specifically).

Using optimization principle (see Supplementary Note 1.1) combined with topology features of metabolic network, we can obtain the following qualitative behavior (agree with elementary flux mode[2, 23]): in the case of Supplementary Fig. 1b, either *A*1 or *A*2 will be utilized depending on the growth rate of individual mediums, yet unable to predict the turning point (or ratio sensing behavior); in the case of Supplementary Fig. 1d, three strategies (using only *A*; using only *B*; or using *A* and *B*) are permitted, yet unable to predict if *A* and *B* would be co-utilized, neither does

the carbon supply percentage in cases of co-utilization.

**Supplementary Note 6.5 Exceptional cases**

On qualitative aspect, there are two exceptional cases of the branch efficiency analysis (Supplementary Note 2.2) and optimal condition no longer corresponds to elementary flux mode: (a) Overlapping enzymes; (b) Enzyme regulations among each other.

Case (a):

Consider the following scheme:

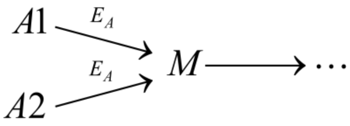


,

where $E_A$, a single enzyme (or transporter) catalyzing two distinct reactions. Although the topology here is similar to that of Supplementary Fig. 1b, $A1$ and $A2$ would be co-utilized when $\varepsilon$ is maximized.

Case (b):

Conceptually, activity of an enzyme is possible to be influenced by other enzymes. Consider the following scheme:

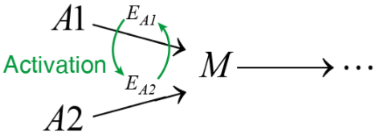


,

$E_{A1}$ and $E_{A2}$ are carrier enzymes of $A1$ and $A2$, respectively. Specifically:

$$\begin{cases} A1 + E_{A1} \underset{d_{A1}}{\overset{a_{A1}}{\rightleftarrows}} E_{A1} \cdot A1 \xrightarrow{k_{A1}^{\text{cat}}([E_{A2}])} E_{A1} + M \\ A2 + E_{A2} \underset{d_{A2}}{\overset{a_{A2}}{\rightleftarrows}} E_{A2} \cdot A2 \xrightarrow{k_{A2}^{\text{cat}}([E_{A1}])} E_{A2} + M \end{cases}.$$

We consider the following imaginary form of $k_{Ai}^{\text{cat}}$ ($i$=1, 2): $k_{A1}^{\text{cat}}\left(\left[E_{A2}\right]\right) = k_{A1}^{\text{L}} + k_{A1}^{\text{H}} \cdot \left[E_{A2}\right]$, $k_{A2}^{\text{cat}}\left(\left[E_{A1}\right]\right) = k_{A2}^{\text{L}} + k_{A2}^{\text{H}}\left[E_{A1}\right]$ ( $k_{Ai}^{\text{L}}, k_{Ai}^{\text{H}} > 0$ and assume $k_{Ai}^{\text{H}} \gg k_{Ai}^{\text{L}}$ ), where enzymes can promote the activities of each other. The flux from $A1$ to $M$ is $v_{A1} = k_{A1}^{\text{cat}}\left(E_{A2}\right)\dfrac{[A1][E_{A1}]}{[A1]+K_{A1}}$ while

the flux from $A2$ to $M$ is $v_{A2}=k_{A2}^{\text{cat}}\left(E_{A1}\right)\frac{[A2][E_{A2}]}{[A2]+K_{A2}}$ (we assume Michaelis–Menten kinetics).

Denote $\frac{[Ai]}{[Ai]+K_{Ai}}$ as $f_{[Ai]}$, which is independent of $E_{Ai}$. Then $v_{A1}=k_{A1}^{\text{cat}}\left(E_{A2}\right)\cdot\left[E_{A1}\right]\cdot f_{[A1]}$, and $v_{A2}=k_{A2}^{\text{cat}}\left(E_{A1}\right)\cdot\left[E_{A2}\right]\cdot f_{[A2]}$ Here, $\Phi_{\text{tot}}=V_{\text{cell}}\cdot\left([E_{A1}]\cdot n_{E_{A1}}+[E_{A2}]\cdot n_{E_{A2}}\right)$, while $J_{\text{tot}}\equiv V_{\text{cell}}\cdot\left(v_{A1}+v_{A2}\right)$. According to Supplementary Equation 1,

$$\varepsilon=\frac{k_{A1}^{\text{L}}\cdot\left[E_{A1}\right]\cdot f_{[A1]}+k_{A2}^{\text{L}}\cdot\left[E_{A2}\right]\cdot f_{[A2]}+\left(k_{A1}^{\text{H}}\cdot f_{[A1]}+k_{A2}^{\text{H}}\cdot f_{[A2]}\right)\cdot\left[E_{A1}\right]\cdot\left[E_{A2}\right]}{[E_{A1}]\cdot n_{E_{A1}}+[E_{A2}]\cdot n_{E_{A2}}}, \quad (32)$$

when $k_{Ai}^{\text{H}}\gg k_{Ai}^{\text{L}}$, at the peaking point of $\varepsilon$, $\left[E_{A1}\right],\left[E_{A2}\right]>0$. In this scenario, the metabolic topology is similar to Supplementary Fig. 1b, yet $A1$ and $A2$ can be co-utilized when $\varepsilon$ is maximized.

## Supplementary Note 7. Summary and discussions on the application scope of our analysis framework

Our analysis framework (Supplementary Note 1, and thus the branch efficiency analysis in Supplementary Note 2.2) is based on irreversible reactions: when $\varepsilon$ is maximized, $\kappa_i\approx k_i$ for intermediate metabolites (observed for most metabolite in *E. coli*[9, 10]), with the knowledge of specific activity (defined as enzyme turnover number divided by molecular weight) of catabolic enzymes, we obtain substrate quality $\kappa_i$ and can predict the growth behavior of microbes on mixed carbon sources. With this framework, we quantitatively explain the phenomenon of diauxie versus co-utilization; predictions of carbon supply percentage in various combinations of mixtures agree well with experimental results.

In Supplementary Note 5, we show that this framework applies to reversible reactions. In Supplementary Note 6, we demonstrate that this framework is broadly applicable to cases such as reversible reactions, enzyme concentration dependent reaction rate, allosteric enzymes, irreversible metabolite inhibitions, and reversible competitive inhibition. When all forms of metabolic regulations are permitted, this framework only qualitatively applies, i.e. qualitatively behavior that is possible to show up based on the metabolic topology (Supplementary Note 6.4). However, conceptually or in practice, there are two exceptional cases for this framework:

Bi-substrates transporters/enzymes (e.g. glucose transporters in *E. coli* can co-transport mannose[25]), and enzymes regulation among each other (Supplementary Note 6.5). Nevertheless, we can consider these effects specifically when they are involves.

Overall, our analysis framework (Supplementary Note 1) can be broadly applicable for microbial studies and useful in quantitatively explaining why and how microbes make the choices when facing multiple carbon sources.

## Supplementary Figures

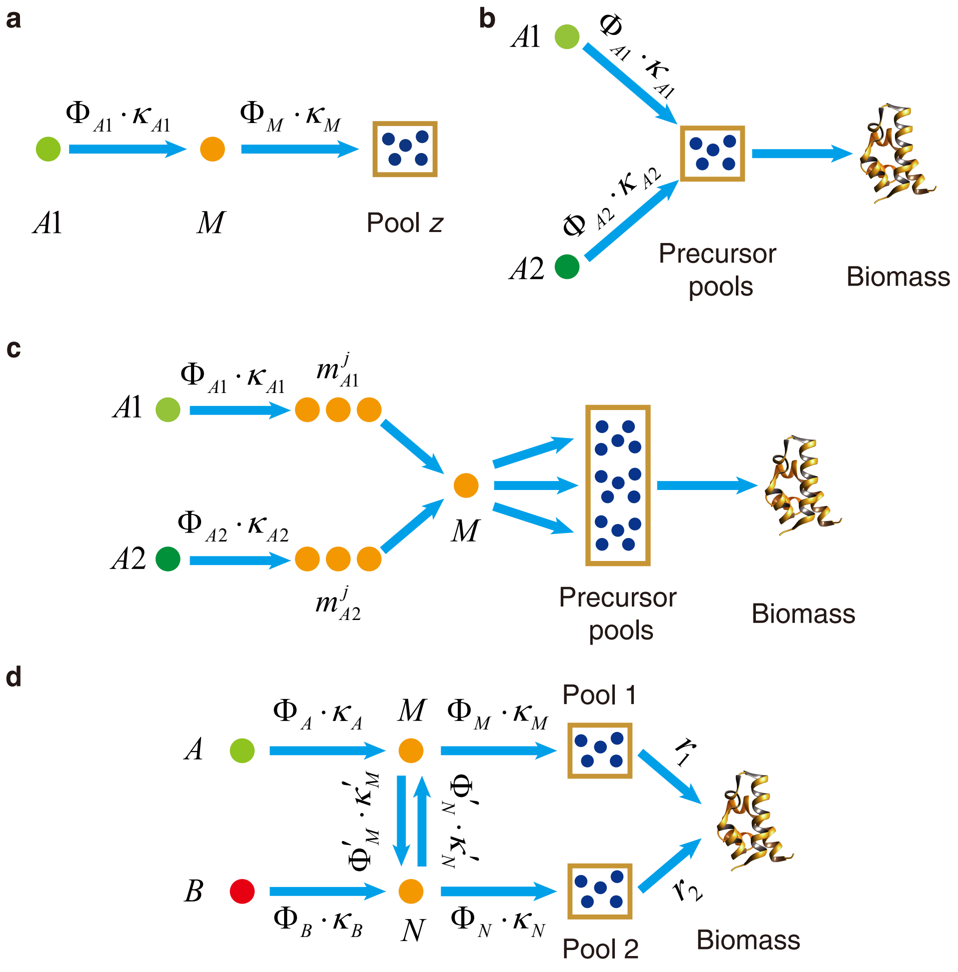


**Supplementary Figure 1. Coarse-grained models of metabolism and biomass production.**
(**a**) A coarse-grained metabolic model with one intermediate node.
(**b**) Minimal model of diauxie. The carbon sources $A1$ or $A2$ or both can supply the precursor pools. The cell grows faster if only the more efficient source is utilized.
(**c**) Topology of metabolic network with two Group A sources. The two carbon flux pathways from sources $A1$ and $A2$ can have multiple intermediate nodes (metabolites) $m^j_{A1}$ and $m^j_{A2}$ before merging to a common node $M$, after which the flux is diverted to various precursor pools.
(**d**) Minimal model of co-utilization. In synthesizing biomass, the two precursor pools supply $r_1$ and $r_2$ carbon flux, respectively. Either pool can draw flux from either of the two sources A and B. Under certain conditions, it is optimal for different sources to supply different pools.

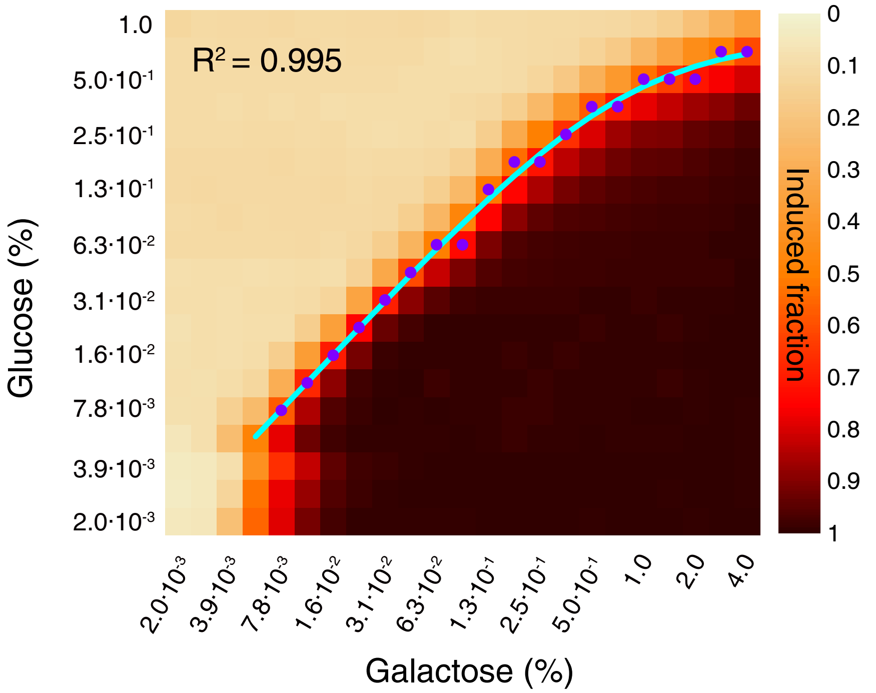


**Supplementary Figure 2. Concentration dependence of turning point.** In the experiment of Escalante-Chong et al.[11], yeast cells were cultured with a mixture of glucose and galactose of various combinations of concentrations. The induction of galactose pathway was measured in single cells with flow cytometry. The heat map represents the fraction of cells with the galactose pathway turned on for given pairs of concentrations (reproduced with permission). The purple dots indicate the glucose concentration at which the induction fraction is at or just above 0.5 for given galactose concentration. The solid line is a fit with Supplementary Equation 14 ($R^2 = 0.995$, $\delta = 0.8256$ and $\Delta = 0.8052$).

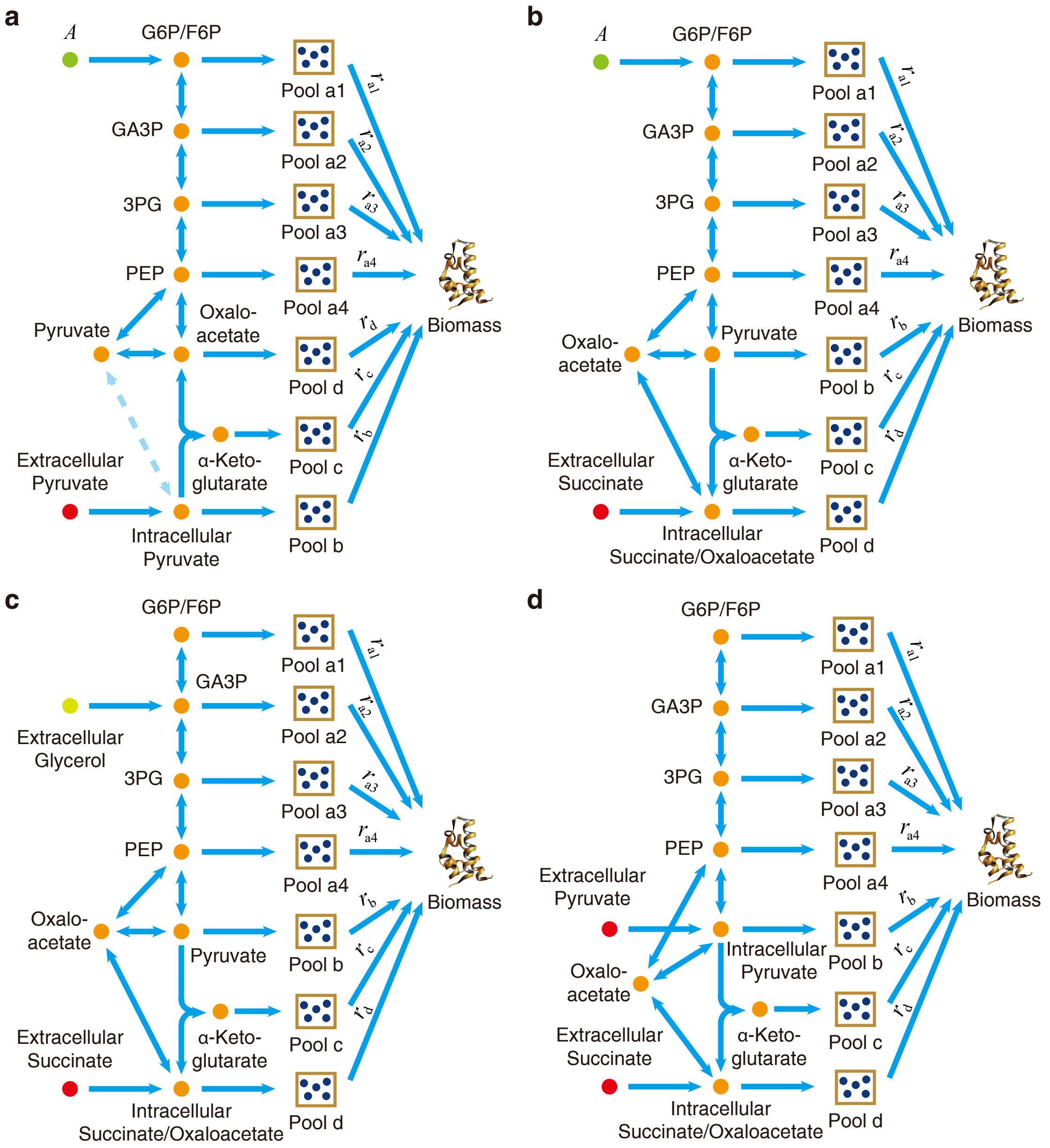


**Supplementary Figure 3. Topology of the metabolic network when a Group B source is mixed with a Group A source or with another Group B source.**

(**a**) Pyruvate (a Group B source) is mixed with a Group A source.

(**b**) Succinate (a Group B source) is mixed with a Group A source.

(**c**) Succinate (a Group B source) is mixed with Glycerol (a Group A source).

(**d**) Pyruvate (a Group B source) is mixed with Succinate (a Group B source).

See Supplementary Note 1.3 for the classifications of Pools a1-a4, b-d.

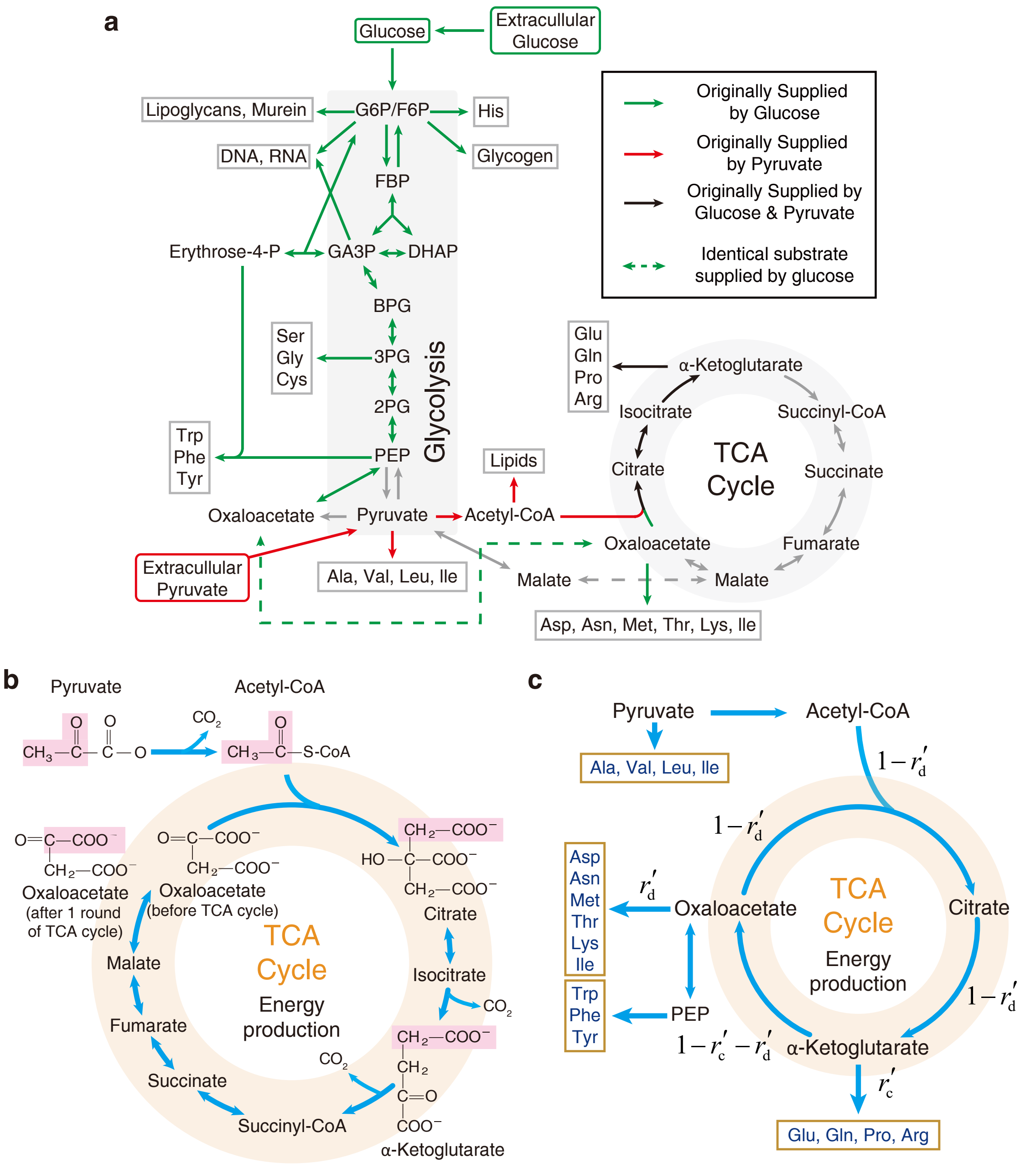


**Supplementary Figure 4. Pool suppliers in the case of co-utilization. (a)** Original pool suppliers (Model predictions, see Supplementary Table3) in the case of glucose-pyruvate (both with saturated concentrations) co-utilization. Metabolites connected by green arrows are originally supplied by glucose, those connected by red arrows are originally supplied by pyruvate, and those connected by black arrows are originally supplied by both glucose and pyruvate. (**b**) & (**c**) Influence of the TCA cycle on the pool suppliers in practice. (**b**) Reactions of the TCA cycle. Through a round of TCA cycle, half of the carbon atoms of an oxaloacetate molecule are replaced

by that of pyruvate (marked with light pink shades). (**c**) Stoichiometry allocation of the carbon flux in TCA cycle. For a given microbial growth rate, we assume the stoichiometry of carbon flux at oxaloacetate is 1 per unit time $\tau$, $r'_{\mathrm{d}}$ stoichiometry of the flux flows to Pool d (Aspartic acid, etc.), $1-r'_{\mathrm{d}}$ stoichiometry of flux flows to citrate, accompanied with the same stoichiometry of carbon flux joined from Citrate. $r'_{\mathrm{c}}$ stoichiometry of the flux flows to Pool c (Glutamic acid, etc.), with $1-r'_{\mathrm{c}}-r'_{\mathrm{d}}$ stoichiometry of carbon flux flows back to oxaloacetate. To keep sustainable microbial growth, $r'_{\mathrm{c}}+r'_{\mathrm{d}}$ stoichiometry of carbon flux would join from the original supplier of oxaloacetate (entry point of Pool d).

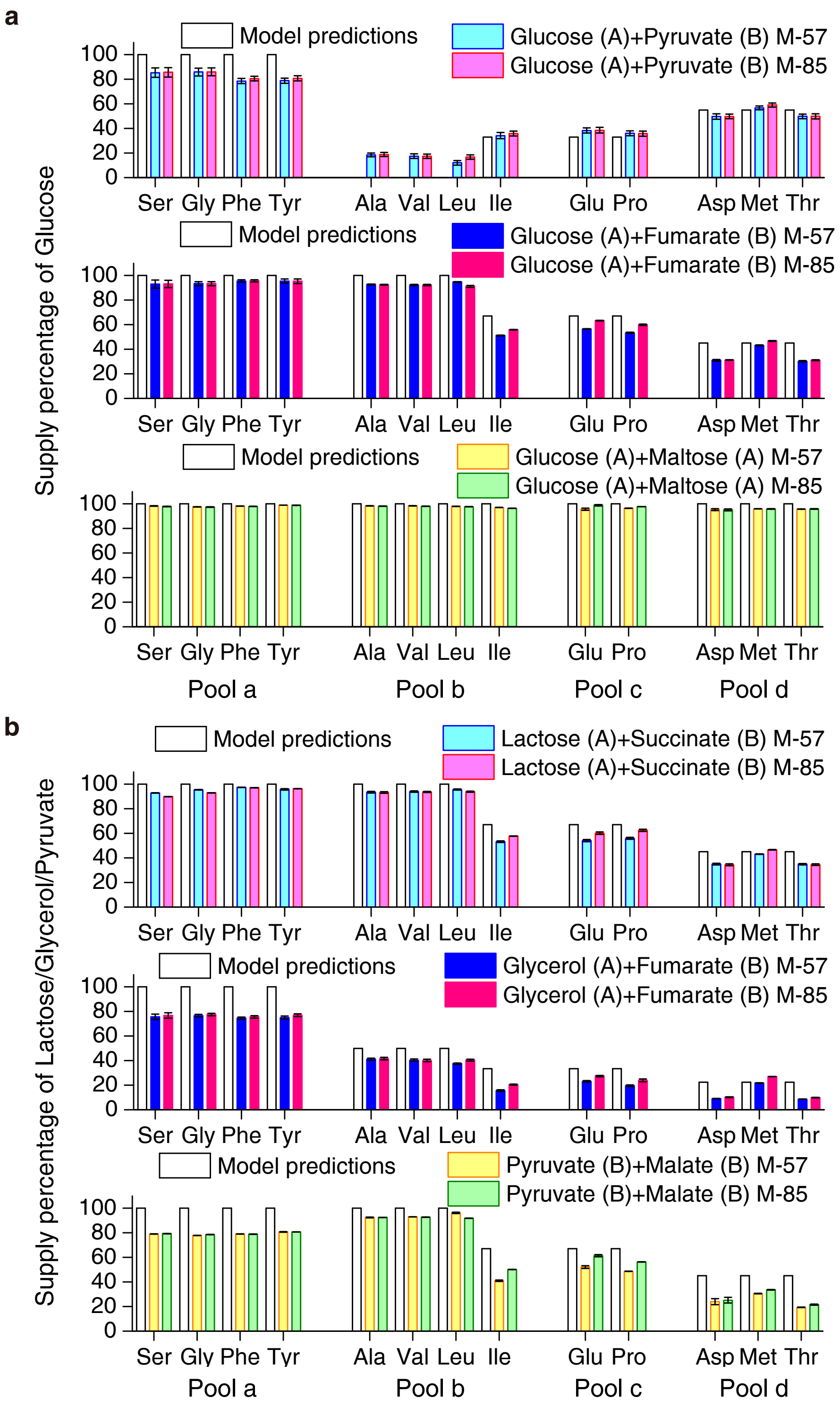


**Supplementary Figure 5. Comparison of pool suppliers determined using experimental data M-57 and M-85. (a)** Cases of glucose mixed with another carbon source. **(b)** Cases of lactose, glycerol or pyruvate mixed with a Group B carbon source. Leu M-15 and Ile M-15 data are used in the M-57 results (see Methods for details). In all cases, there is no significant difference

between results obtained using data M-57 and data M-85. Model predictions are marked with hollow bars while experimental results are marked with color bars. Error bars represent standard deviations. Source data are provided as a Source Data file.

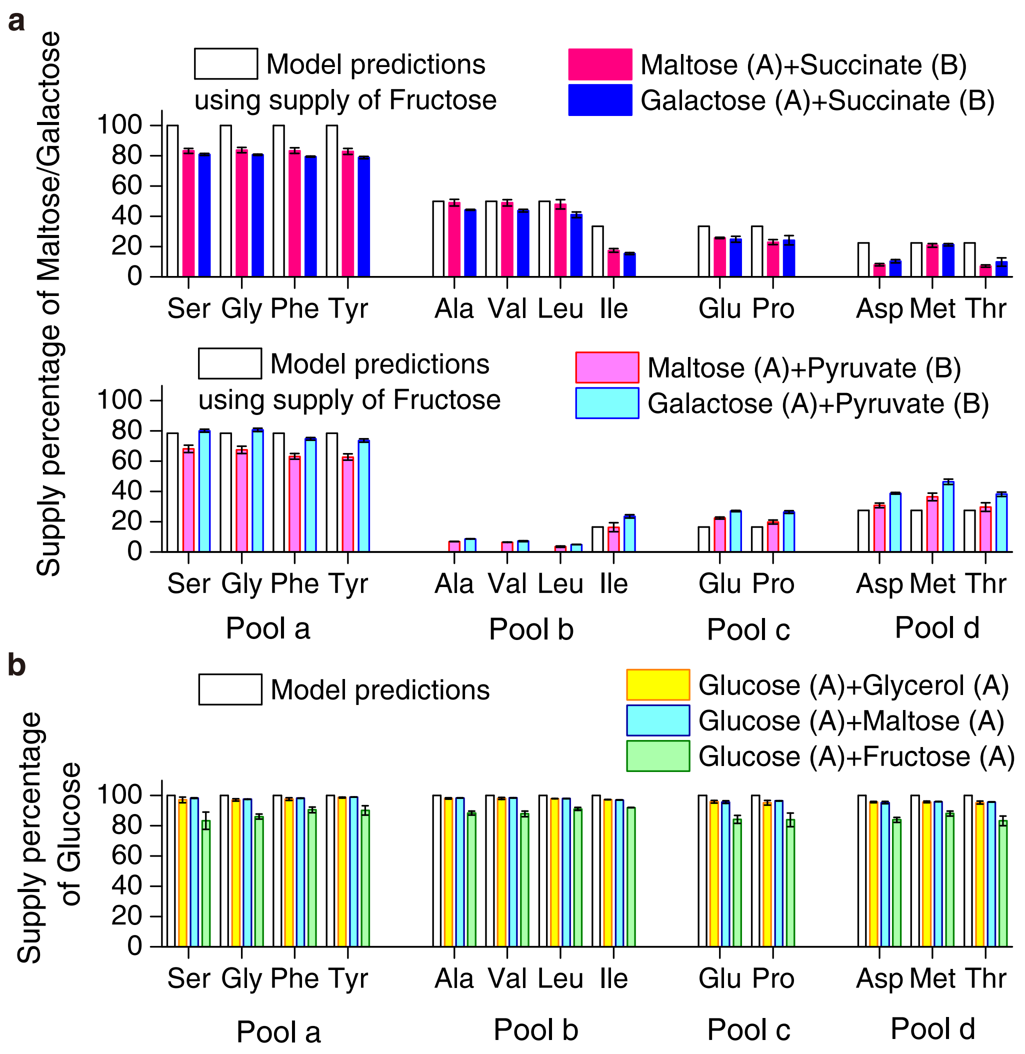


**Supplementary Figure 6. Suppliers of precursor pools on mixed carbon sources. (a)** Cases of co-utilization (A+B). Here the supply percentage of maltose and galactose in each case are compared with model predictions using supply of fructose (Fructose+Succinate or Fructose+Pyruvate), since some biochemical parameters of the catabolic enzymes of maltose, and galactose are unable to find, while the topology and supply percentage are quite similar to that of fructose. (**b**) Cases of diauxie (A+A): glucose mixed with glycerol, maltose, and fructose. For glucose mixed with glycerol and maltose (non-PTS sugars), all pools are supplied by glucose, which are perfect cases of diauxie. For glucose mixed with fructose (a PTS sugar), the majority of carbon (>83%) in all pools are supplied by glucose, with small portions supplied by fructose, which might due to imperfect molecular inhibition. Model predictions are marked with hollow bars while experimental results are marked with color bars. Error bars represent standard deviations. Source data are provided as a Source Data file.

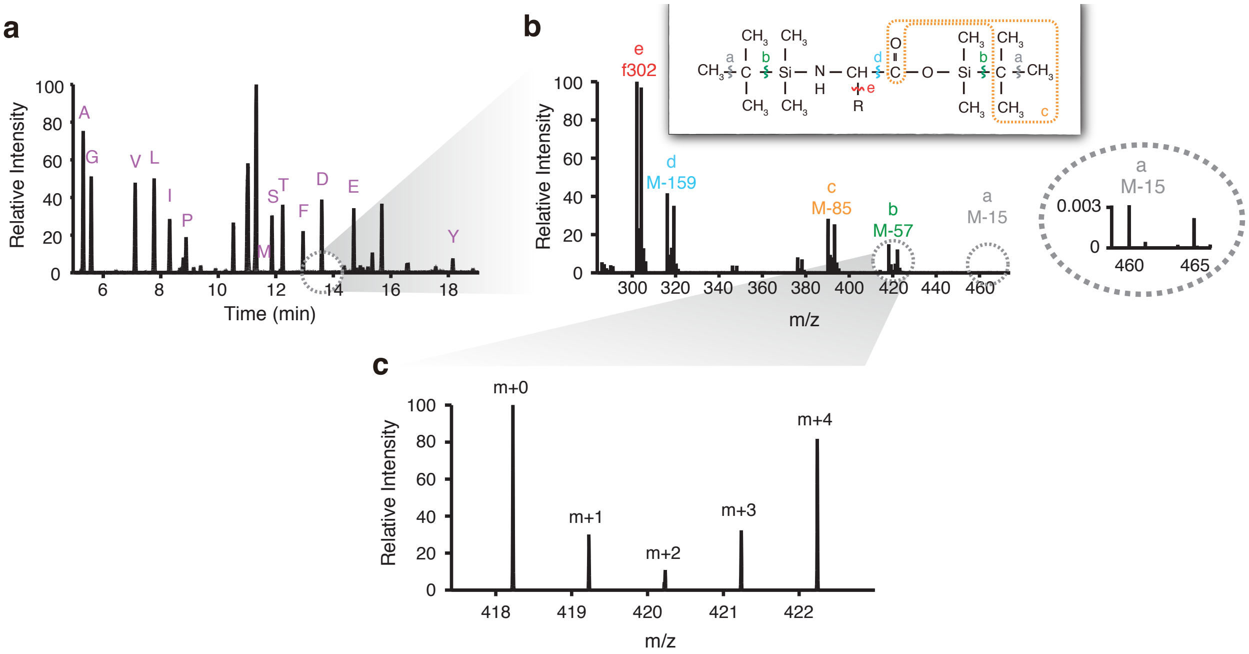


**Supplementary Figure 7. Measurement of $^{13}$C labeling percentage by GC-MS analysis[26, 27].** (**a**) The gas chromatogram of derivatized amino acid from *E.coli* hydrolysate, annotated by their one-letter abbreviation. These different amino acids were separated by GC according to their different retention time. Inset of (**b**) The structure of derivatized amino acids. During ionization, the derivatized amino acids were fragmented, either cracking at the wave line or losing the atoms in the box, resulting in various fragments, including a: M-15, b: M-57, c: M-85, d: M-159 and e: f302. These different fragments of the same amino acid were further separated and analyzed by MS. (**b**) Integrated mass spectrum over the full GC peak of derivatized aspartic acid (retention time from 13.57~13.63 min). There are five different fragments of derivatized aspartic acid detected by the MS. For every fragment, several mass isotopomer peaks are detected, referring to the same chemical structure but incorporating different number of $^{13}$C atoms. (**c**). The mass spectrum of M-57 fragment of derivatized aspartic acid. There are five mass isotopomer peaks of M-57 fragment. m + 0 denotes the mass isotopomer that contains no $^{13}$C atoms. m + 1, m + 2, m + 3 and m + 4 denote the mass isotopomer that contains 1 to 4 $^{13}$C atoms respectively.

## Supplementary Tables

**Supplementary Table 1. $k_{cat}$ and molecular weight (MW) reference data of *E. coli*.**

| Reaction | Enzyme/ Transporter | $k_{cat}$ ($s^{-1}$) | MW (kDa) | References |
|---|---|---|---|---|
| Glucose → Glucose-6P | Glucokinase | $4.1\times10^2$ | $7.0\times10$ | 28-30 |
| Glucose-6P ↔ Fructose-6P | Glucose-6-phosphate isomerase | $2.6\times10^2$ | $1.2\times10^2$ | 31, 32 |
| Fructose-6P → Fructose-1,6P | Phosphofructokinase | $4.4\times10^2$ | $1.4\times10^2$ | 33, 34 |
| Fructose-1,6P→Fructose-6P | Fructose-1,6-bisphosphatase | $2.0\times10$ | $3.6\times10$ | 35, 36 |
| Fructose-1,6P ↔ Glyceraldehyde 3-phosphate+ Dihydroxyacetone phosphate | Fructose-bisphosphate aldolase | $1.4\times10$ | $7.8\times10$ | 37, 38 |
| Dihydroxyacetone phosphate ↔ Glyceraldehyde 3-phosphate | Triosephosphate Isomerase | $4.3\times10^2$ | $5.4\times10$ | 39, 40 |
| Glyceraldehyde 3-phosphate ↔ 1,3-Bisphosphoglycerate | Glyceraldehyde-3-ph-osphate dehydrogenase | $9.5\times10$ | $1.4\times10^2$ | 41, 42 |
| 1,3-Bisphosphoglycerate ↔ 3-Phosphoglycerate | Phosphoglycerate kinase | $3.5\times10^2$ | $4.4\times10$ | 43, 44 |
| 3-Phosphoglycerate ↔ 2-Phosphoglycerate | Phosphoglycerate mutase | $3.3\times10^2$ | $4.9\times10$ | 45 |
| 2-Phosphoglycerate ↔ Phosphoenolpyruvate | Enolase | $2.2\times10^2$ | $9.0\times10$ | 46, 47 |
| Phosphoenolpyruvate → Pyruvate | Pyruvate kinase | $5.0\times10^2$ | $2.4\times10^2$ | 48 |
| Pyruvate →Acetyl-CoA | Pyruvate dehydrogenase | $1.2\times10^2$ | $1.0\times10^2$ | 49 |
| Oxaloacetate+Acetyl-CoA → Citrate | Citrate synthase | $2.4\times10^2$ | $9.7\times10$ | 50, 51 |
| Citrate ↔ Isocitrate | Aconitate hydratase | $7.0\times10$ | $9.4\times10$ | 52, 53 |
| Isocitrate→ α-Ketoglutarate | Isocitrate dehydrogenase | $2.0\times10^2$ | $9.5\times10$ | 42, 54, 55 |
| α-Ketoglutarate → Succinyl-CoA | α-Ketoglutarate dehydrogenase complex E1 component | $1.5\times10^2$ | $1.9\times10^2$ | 56, 57 |
| Succinyl-CoA ↔ Succinate | Succinyl-CoA synthetase | $9.1\times10$ | $1.6\times10^2$ | 58 |
| Succinate → Fumarate | Succinate dehydrogenase | $1.1\times10^2$ | $1.0\times10^2$ | 59, 60 |
| Fumarate →Succinate | Fumarate reductase | $2.5\times10^2$ | $9.3\times10$ | 59, 61 |
| Fumarate ↔ Malate | Fumarase | $1.2\times10^3$ | $2.0\times10^2$ | 62, 63 |
| Malate ↔ Oxaloacetate | Malate dehydrogenase | $5.5\times10^2$ | $6.1\times10$ | 64 |
| Phosphoenolpyruvate → Oxaloacetate | Phosphoenolpyruvate carboxylase | $1.5\times10^2$ | $4.0\times10^2$ | 65, 66 |
| Oxaloacetate → Phosphoenolpyruvate | Phosphoenolpyruvate carboxykinase | 4.3 | $6.0\times10$ | 67-69 |
| Malate → Pyruvate | Malic enzyme | $8.3\times10$ | $2.7\times10^2$ | 70, 71 |
| Pyruvate →Malate | Malic enzyme | 2.9 | $2.7\times10^2$ | 70, 71 |
| Pyruvate → Oxaloacetate | - | - | | - |
| Pyruvate → Phosphoenolpyruvate | Pyruvate, water dikinase | $3.5\times10$ | $2.5\times10^2$ | 72 |
| Extracellular Glucose → Glucose-6P | Glucose-specific PTS enzyme | $1\times10^2$ | $5.0\times10$ | 12, 73-76 |
| Lactose membrane transport | Lactose permease | $5\times10$ | $4.6\times10$ | 77, 78 |

| | | | | |
|---|---|---|---|---|
| Lactose→Glucose+Galactose | β-galactosidase | $6.4\times10^2$ | $4.6\times10^2$ | Estimated [79, 80] |
| Extracellular Fructose →Fructose-6P† | Fructose-specific PTS enzyme | 17 | $5.8\times10$ | Estimated [12, 76] |
| Glycerol membrane transport | Glycerol facilitator | 4 | $2.5\times10$ | Estimated [12, 81-83] |
| Glycerol →Glycerol-3-phosphate | Glycerol kinase | $1.4\times10^2$ | $2.1\times10^2$ | [84, 85] |
| Glycerol-3-phosphate ↔ Dihydroxyacetone phosphate | Glycerol-3-phosphate dehydrogenase | $6.8\times10$ | $1.1\times10^2$ | [86] |
| Pyruvate membrane transport | Pyruvate transporter | 9 | $4\times10$ | Estimated [12, 42, 87-89] |
| Oxaloacetate membrane transport | Oxaloacetate transporter | 5 | $4\times10$ | Estimated [12, 42, 90-92] |
| Succinate membrane transport | Succinate transporter | 5.1* | $4\times10$ | Estimated [12, 42, 92-94] |
| Fumarate membrane transport | Fumarate transporter | 4.6* | $4\times10$ | Estimated [12, 42, 92, 95] |
| Malate membrane transport | Malate transporter | 4.5* | $4\times10$ | Estimated [12, 42, 92, 95] |

† The uptake of extracellular fructose is different (Extracellular Fructose→Fructose-1P) at low concentration (<2mM).

* Estimated value around 5 with fitting decimal digits since that the growth rate of *E.coli* are similar in culturing medium with succinate, fumarate or malate as the single carbon source (see Supplementary Table 5).

**Supplementary Table 2. Substrate branch efficiency of Pools a1-a4, Pool b and Pool d of *E. coli*.** (With enzyme MW normalization unit 100 kDa, branch efficiency unit $s^{-1}$, and carbon sources of saturated concentrations.)

| **Substrate (Sub)** | **Pool a1** | **Pool a2** | **Pool a3** | **Pool a4** | **Pool b** | **Pool d** |
|---|---|---|---|---|---|---|
| | Ser, Gly, Cys, Trp, Phe, Tyr, His; precursors of RNA, DNA, Glycogen, Lipoglycans, Murein. | | | | Ala, Val, Leu, Ile[†];precursors of Lipids. | Asp, Asn, Met, Thr, Lys, Ile[‡] |
| | $\varepsilon_{\text{Sub}\to\text{F6P}}$ | $\varepsilon_{\text{Sub}\to\text{GA3P}}$ | $\varepsilon_{\text{Sub}\to\text{3PG}}$ | $\varepsilon_{\text{Sub}\to\text{PEP}}$ | $\varepsilon_{\text{Sub}\to\text{pyruvate}}$ | $\varepsilon_{\text{Sub}\to\text{oxaloacetate}}$ |
| Glucose | 104 | 14.6 | 11.7 | 10.9 | 10.4 | 8.5 |
| Lactose | 44 | 12.3 | 10.1 | 9.6 | 9.2 | 7.6 |
| Fructose | 29 | 10.8 | 9.1 | 8.6 | 8.3 | 7.0 |
| Glycerol | 5.9 | 10.5 | 9.0 | 8.6 | 8.2 | 7.0 |
| Pyruvate | 4.7 | 7.3 | 8.2 | 8.6 | 22.5 | 7.0 |
| [*]Oxaloacetate | 3.6 | 4.8 | 5.2 | 5.4 | 8.8 | 12.5 |
| Malate | 3.5 | 4.7 | 5.0 | 5.2 | 8.2 | 11.1 |
| Fumarate | 3.5 | 4.7 | 5.0 | 5.2 | 8.2 | 11.1 |
| Succinate | 3.5 | 4.7 | 5.0 | 5.2 | 8.2 | 11.1 |

[*]Oxaloacetate can quickly decompose to pyruvate and $CO_2$ spontaneously[16] in solution (Supplementary Note 4.7), which causes the pool suppliers in cases involving oxaloacetate different from that predicted using the branch efficiency.

[†]Isoleucine is supplied by joint fluxes from pyruvate and oxaloacetate (Supplementary Note 4.2).

**Supplementary Table 3. Predicted original pool suppliers of *E. coli* in the cases of co-utilization.** The original suppliers of Pools a, b, d are determined by the branch efficiency listed in Supplementary Table 2. Owing to the Influence of TCA cycle, the pool supplier in practice can be different from its original supplier.

| **No.** | **Mixed Substrates** | **Pools a1, a2, a3, a4** | **Pool b** | **Pool d** |
|---|---|---|---|---|
| | | Ser, Gly, Cys, Trp, Phe, Tyr, His; precursors of RNA, DNA, Glycogen, Lipoglycans, Murein. | Ala, Val, Leu, Ile; precursors of Lipids | Asp, Asn, Met, Thr, Lys, Ile |
| | | **Original Supplier** | **Original Supplier** | **Original Supplier** |
| **1** | Glucose/ Lactose + Pyruvate | Glucose/ Lactose | Pyruvate | Glucose/ Lactose |
| **2** | Glucose/ Lactose + Succinate/ Malate/ Fumarate | Glucose/ Lactose | Glucose/ Lactose | Succinate/ Malate/ Fumarate |
| **3** | Fructose/Glycerol + Pyruvate | Fructose/Glycerol (a1, a2, a3, 50%[*] a4), Pyruvate (50%[*] a4) | Pyruvate | Fructose/Glycerol (50%[*]), Pyruvate (50%[*]) |
| **4** | Fructose/Glycerol +Succinate/ Malate/ Fumarate | Fructose/Glycerol | Fructose/Glycerol (50%[*]), Succinate/ Malate/ Fumarate (50%[*]) | Succinate/ Malate/ Fumarate |
| **5** | Pyruvate +Succinate/ Malate/ Fumarate | Pyruvate | Pyruvate | Succinate/ Malate/ Fumarate |
| **6** | Succinate +Malate[†] | Succinate(50%[*]), Malate(50%[*]) | Succinate(50%[*]), Malate(50%[*]) | Succinate(50%[*]), Malate(50%[*]) |

[*]Roughly identical branch efficiencies from both substrates.
[†]Succinate and malate own shared membrane transporters.

**Supplementary Table 4. Pool suppliers of *E. coli* predicted to be observed in the cases of co-utilization under aerobic conditions.** The pool suppliers in practice are influence by joint fluxes as well as the TCA cycle (see Supplementary Notes 4.1-4.5 for details).

| **No.** | **Mixed Substrates** | **Pools a1, a2, a3, a4** | **Pool b** | **Pool c** | **Pool d** |
|---|---|---|---|---|---|
| | | Ser[†], Gly[†], Cys[†], Trp, Phe, Tyr, His; precursors of RNA, DNA, Glycogen, Lipoglycans, Murein. | Ala, Val, Leu, Ile[*]; precursors of Lipids | Glu, Gln, Pro, Arg | Asp, Asn, Met, Thr, Lys, Ile[*] |
| | | **Suppliers** | **Suppliers** | **Suppliers** | **Suppliers** |
| **1** | Glucose/ Lactose + Pyruvate | Glucose/ Lactose | Pyruvate | Glucose/ Lactose (33%), Pyruvate (67%) | Glucose/ Lactose (55%), Pyruvate (45%) |
| **2** | Glucose/ Lactose + Succinate/ Malate/ Fumarate | Glucose/ Lactose | Glucose/ Lactose | Glucose/ Lactose (67%), Succinate/ Malate/ Fumarate (33%) | Glucose/ Lactose (45%), Succinate/ Malate/ Fumarate (55%) |
| **3** | Fructose/Glycerol + Pyruvate | Fructose/Glycerol (a1, a2, a3, 50% a4) , Pyruvate (50% a4) | Pyruvate | Fructose/Glycerol (16.5%), Pyruvate (83.5%) | Fructose/Glycerol (27.5%), Pyruvate (72.5%) |
| **4** | Fructose/Glycerol +Succinate/ Malate/ Fumarate | Fructose/Glycerol | Fructose/Glycerol (50%), Succinate/ Malate/ Fumarate (50%) | Fructose/Glycerol (33.5%), Succinate/ Malate/ Fumarate (66.5%) | Fructose/Glycerol (22.5%), Succinate/ Malate/ Fumarate (77.5%) |
| **5** | Pyruvate, Succinate/ Malate/ Fumarate | Pyruvate | Pyruvate | Pyruvate (67%), Succinate/ Malate/ Fumarate (33%) | Pyruvate (45%), Succinate/ Malate/ Fumarate (55%) |
| **6** | Succinate, Malate | Succinate(50%), Malate(50%) | Succinate(50%), Malate(50%) | Succinate(50%), Malate(50%) | Succinate(50%), Malate(50%) |

[†]The supply percentage of serine, glycine and cysteine can be affected by the reversible reactions among the precursors of Pool a2-a4 (Supplementary Note 5.1).

[*] 2/5 of carbon atoms in isoleucine in practice supplied by Pools b and 3/5 of carbon atoms in practice supplied by Pools d (Supplementary Note 4.2).

**Supplementary Table 5. Exponential growth rate of *E. coli* NCM3722.**

| Growth rate†($h^{-1}$) | alone | glucose | lactose | glycerol | fructose | maltose | galactose | pyruvate | succinate |
|---|---|---|---|---|---|---|---|---|---|
| alone | / | 1.51†† | 1.54†† | 1.04 | 1.11 | 1.18 | 0.83* | 1.15 | 1.07 |
| pyruvate | 1.15 | 1.38** | 1.58 | 1.45 | 0.99** | 1.21 | 1.40 | / | 1.37 |
| succinate | 1.07 | 1.57 | 1.57 | 1.32 | 1.31 | 1.23 | 1.23 | 1.37 | / |
| fumarate | 1.03 | 1.55 | 1.53 | 1.31 | 1.28 | 1.20 | 1.17 | 1.32 | 0‡ |
| malate | 1.02 | 1.53 | 1.54 | 1.30 | 1.32 | 1.26 | 1.23 | 1.34 | 0.98 |

†Observational error and standard deviation of the growth rate data around or less than $0.07h^{-1}$. All numbers are averages over three independent experiments. Source data are provided as a Source Data file.

*The growth rate of *E.coli* in galactose alone medium is suboptimal due to *GalS* regulation[96].

**Membrane transport of Phosphotransferase System (PTS) sugars (such as glucose and fructose) are coupled with the conversion from PEP to pyruvate[97]. This might lead to suboptimal growth when PTS sugars mixed with pyruvate.

††Growth rate of *E.coli* is a bit higher in lactose alone medium than that of glucose (although quite similar, and similar in mixed medium). The existence of *lac* operon might due to that glucose is more wide spread in natural environment.

‡ *E.coli* did not grow in succinate+fumarate and malate +fumarate culture medium in 72 hours.

**Supplementary Table 6. All possible patterns of the original pool suppliers of *E. coli* in mixed carbon sources.** Here we consider all possibilities for the choice of the biochemical parameters ($k_{cat}$ can be any positive values). Predictions based on parameters listed in Supplementary Table 1 are shown in blue text (see Supplementary Table 3 for details).

| Mixed Substrates | Possible Pattern Types | Pools a1, a2, a3, a4 | Pool b | Pool d |
|---|---|---|---|---|
| | | Ser, Gly, Cys, Trp, Phe, Tyr, His; precursors of RNA, DNA, Glycogen, Lipoglycans, Murein. | Ala, Val, Leu, Ile; precursors of Lipids | Asp, Asn, Met, Thr, Lys, Ile |
| | | Original Supplier | Original Supplier | Original Supplier |
| A (Glucose/ Lactose/ Fructose/ Glycerol/ Maltose/ Galactose) + B1 (Pyruvate) | DX* No.1 | A | A | A |
| | DX No.2 | B1 | B1 | B1 |
| | CoU* No.1 | A | B1 | A |
| | CoU No.2 | A | B1 | B1 |
| | CoU No.3 | A (a1, a2, a3), B1 (a4) | B1 | B1 |
| | CoU No.4 | A (a1, a2), B1 (a3, a4) | B1 | B1 |
| | CoU No.5 | A(a1), B1 (a2, a3, a4) | B1 | B1 |
| | CoU No.6 | A | A (50%), B1 (50%) | A |
| | CoU No.7 | A | A (50%), B1 (50%) | A (50%), B1 (50%) |
| | CoU No.8 | A | B1 | A (50%), B1 (50%) |
| | CoU No.9 | A (a1, a2, a3, 50% a4), B1 (50% a4) | B1 | A (50%), B1 (50%) |
| | CoU No.10 | A (a1, a2, a3, 50% a4), B1 (50% a4) | B1 | B1 |
| | CoU No.11 | A (a1, a2, 50% a3), B1 (a4, 50% a3) | B1 | B1 |
| | CoU No.12 | A (a1, 50% a2), B1 (a3, a4, 50% a2) | B1 | B1 |
| | CoU No.13 | A (50% a1), B1 (a2, a3, a4, 50% a1) | B1 | B1 |
| A (Glucose/ Lactose/ Fructose/ Glycerol/ Maltose/ Galactose) +B2 (Succinate/ | DX No.1 | A | A | A |
| | DX No.2 | B2 | B2 | B2 |
| | CoU No.1 | A | A | B2 |
| | CoU No.2 | A | B2 | B2 |
| | CoU No.3 | A | B2 | A |
| | CoU No.4 | A (a1, a2, a3), B2 (a4) | B2 | B2 |
| | CoU No.5 | A (a1, a2), B2 (a3, a4) | B2 | B2 |
| | CoU No.6 | A (a1), B2 (a2, a3, a4) | B2 | B2 |

| | | | | |
|---|---|---|---|---|
| Malate/ Fumarate) | CoU No.7 | A | A (50%), B2 (50%) | A |
| | CoU No.8 | A | A | A (50%), B2 (50%) |
| | CoU No.9 | A | A (50%), B2 (50%) | B2 |
| | CoU No.10 | A | B2 | A (50%), B2 (50%) |
| | CoU No.11 | A | A (50%), B2 (50%) | A (50%), B2 (50%) |
| | CoU No.12 | A (a1, a2, a3, 50% a4), B2 (50% a4) | B2 | B2 |
| | CoU No.13 | A (a1, a2, a3, 50% a4), B2 (50% a4) | A (50%), B2 (50%) | B2 |
| | CoU No.14 | A (a1, a2, a3, 50% a4), B2 (50% a4) | B2 | A (50%), B2 (50%) |
| | CoU No.15 | A (a1, a2, 50% a3), B2 (a4, 50% a3) | B2 | B2 |
| | CoU No.16 | A (a1, 50% a2), B1 (a3, a4, 50% a2) | B2 | B2 |
| | CoU No.17 | A (a1, 50% a2), B1 (a3, a4, 50% a2) | B2 | B2 |
| B1 (Pyruvate) +B2 (Succinate/ Malate/ Fumarate) | DX No.1 | B1 | B1 | B1 |
| | DX No.2 | B2 | B2 | B2 |
| | CoU No.1 | B1 | B1 | B2 |
| | CoU No.2 | B2 | B1 | B2 |
| | CoU No.3 | B1 | B1 | B1 (50%), B2 (50%) |
| | CoU No.4 | B2 | B1 (50%), B2 (50%) | B2 |
| | CoU No.5 | B1 (50%), B2 (50%) | B1 | B2 |
| | CoU No.6 | B1 (50%), B2 (50%) | B1 (50%), B2 (50%) | B2 |
| | CoU No.7 | B1 (50%), B2 (50%) | B1 | B1 (50%), B2 (50%) |
| | CoU No.8 | B1 (50%), B2 (50%) | B1 (50%), B2 (50%) | B1 (50%), B2 (50%) |
| Succinate + Malate | DX No.1 | Succinate | Succinate | Succinate |
| | DX No.2 | Malate | Malate | Malate |
| | CoU No.1 | Succinate (50%), Malate (50%) | Succinate (50%), Malate (50%) | Succinate (50%), Malate (50%) |

*DX denotes diauxie and CoU signifies co-utilization.

**Supplementary Table 7. All possible patterns of the pool suppliers of *E. coli* under aerobic conditions in mixed carbon sources.** Here we consider all possibilities for the choice of the biochemical parameters ($k_{cat}$ can be any positive values). Predictions based on parameters listed in Supplementary Table 1 are shown in blue text (see Supplementary Table 4 for details). Other patterns that qualitatively similar with experiments are shown in orange text.

| Mixed Substrates | Possible Pattern Types | Pools a1, a2, a3, a4 | Pool b | Pool c | Pool d |
|---|---|---|---|---|---|
| | | Ser, Gly, Cys, Trp, Phe, Tyr, His; precursors of RNA, DNA, Glycogen, Lipoglycans, Murein. | Ala, Val, Leu, Ile*; precursors of Lipids | Glu, Gln, Pro, Arg | Asp, Asn, Met, Thr, Lys, Ile* |
| | | **Suppliers** | **Suppliers** | **Suppliers** | **Suppliers** |
| A (Glucose/ Lactose/ Fructose/ Glycerol/ Maltose/ Galactose) + B1 (Pyruvate) | DX* No.1 | A | A | A | A |
| | DX No.2 | B1 | B1 | B1 | B1 |
| | CoU* No.1 | A | B1 | A (33%), B1 (67%) | A (55%), B1 (45%) |
| | CoU No.2 | A | B1 | B1 | B1 |
| | CoU No.3 | A (a1, a2, a3), B1 (a4) | B1 | B1 | B1 |
| | CoU No.4 | A (a1, a2), B1 (a3, a4) | B1 | B1 | B1 |
| | CoU No.5 | A(a1), B1 (a2, a3, a4) | B1 | B1 | B1 |
| | CoU No.6 | A | A (50%), B1 (50%) | A (66.5%), B1 (33.5%) | A (77.5%), B1 (22.5%) |
| | CoU No.7 | A | A (50%), B1 (50%) | A (50%), B1 (50%) | A (50%), B1 (50%) |
| | CoU No.8 | A | B1 | A (16.5%), B1 (83.5%) | A (27.5%), B1 (72.5%) |
| | CoU No.9 | A (a1, a2, a3, 50% a4), B1 (50% a4) | B1 | A (16.5%), B1 (83.5%) | A (27.5%), B1 (72.5%) |
| | CoU No.10 | A (a1, a2, a3, 50% a4), B1 (50% a4) | B1 | B1 | B1 |
| | CoU No.11 | A (a1, a2, 50% a3), B1 (a4, 50% a3) | B1 | B1 | B1 |
| | CoU No.12 | A (a1, 50% a2), B1 (a3, a4, 50% a2) | B1 | B1 | B1 |
| | CoU No.13 | A (50% a1), B1 (a2, a3, a4, 50% a1) | B1 | B1 | B1 |
| A (Glucose/ Lactose/ Fructose/ | DX No.1 | A | A | A | A |
| | DX No.2 | B2 | B2 | B2 | B2 |
| | CoU No.1 | A | A | A (67%), B2 (33%) | A (45%), B2 (55%) |

| | | | | | |
|---|---|---|---|---|---|
| Glycerol/ Maltose/ Galactose) +B2 (Succinate/ Malate/ Fumarate) | CoU No.2 | A | B2 | B2 | B2 |
| | CoU No.3 | A | B2 | A (33%), B2 (67%) | A (55%), B2 (45%) |
| | CoU No.4 | A (a1, a2, a3), B2 (a4) | B2 | B2 | B2 |
| | CoU No.5 | A (a1, a2), B2 (a3, a4) | B2 | B2 | B2 |
| | CoU No.6 | A (a1), B2 (a2, a3, a4) | B2 | B2 | B2 |
| | CoU No.7 | A | A (50%), B2 (50%) | A (66.5%), B2 (33.5%) | A (77.5%), B2 (22.5%) |
| | CoU No.8 | A | A | A (83.5%), B2 (16.5%) | A (72.5%), B2 (27.5%) |
| | CoU No.9 | A | A (50%), B2 (50%) | A (33.5%), B2 (66.5%) | A (22.5%), B2 (77.5%) |
| | CoU No.10 | A | B2 | A (16.5%), B2 (83.5%) | A (27.5%), B2 (72.5%) |
| | CoU No.11 | A | A (50%), B2 (50%) | A (50%), B2 (50%) | A (50%), B2 (50%) |
| | CoU No.12 | A (a1, a2, a3, 50% a4), B2 (50% a4) | B2 | B2 | B2 |
| | CoU No.13 | A (a1, a2, a3, 50% a4), B2 (50% a4) | A (50%), B2 (50%) | A (33.5%), B2 (66.5%) | A (22.5%), B2 (77.5%) |
| | CoU No.14 | A (a1, a2, a3, 50% a4), B2 (50% a4) | B2 | A (16.5%), B2 (83.5%) | A (27.5%), B2 (72.5%) |
| | CoU No.15 | A (a1, a2, 50% a3), B2 (a4, 50% a3) | B2 | B2 | B2 |
| | CoU No.16 | A (a1, 50% a2), B1 (a3, a4, 50% a2) | B2 | B2 | B2 |
| | CoU No.17 | A (a1, 50% a2), B1 (a3, a4, 50% a2) | B2 | B2 | B2 |
| B1 (Pyruvate) +B2 (Succinate/ Malate/ Fumarate) | DX No.1 | B1 | B1 | B1 | B1 |
| | DX No.2 | B2 | B2 | B2 | B2 |
| | CoU No.1 | B1 | B1 | B1 (67%), B2 (33%) | B1 (45%), B2 (55%) |
| | CoU No.2 | B2 | B1 | B1 (67%), B2 (33%) | B1 (45%), B2 (55%) |
| | CoU No.3 | B1 | B1 | B1 (83.5%), B2 (16.5%) | B1 (72.5%), B2 (27.5%) |
| | CoU No.4 | B2 | B1 (50%) B2 (50%) | B1 (33.5%), B2 (66.5%) | B1 (22.5%), B2 (77.5%) |
| | CoU No.5 | B1 (50%), B2 (50%) | B1 | B1 (67%), B2 (33%) | B1 (45%), B2 (55%) |

| | | | | | |
|---|---|---|---|---|---|
| | CoU No.6 | B1 (50%), B2 (50%) | B1 (50%), B2 (50%) | B1 (33.5%), B2 (66.5%) | B1 (22.5%), B2 (77.5%) |
| | CoU No.7 | B1 (50%), B2 (50%) | B1 | B1 (83.5%), B2 (16.5%) | B1 (72.5%), B2 (27.5%) |
| | CoU No.8 | B1 (50%), B2 (50%) | B1 (50%), B2 (50%) | B1 (50%), B2 (50%) | B1 (50%), B2 (50%) |
| Succinate + Malate | DX No.1 | Succinate | Succinate | Succinate | Succinate |
| | DX No.2 | Malate | Malate | Malate | Malate |
| | CoU No.1 | Succinate (50%), Malate (50%) | Succinate (50%), Malate (50%) | Succinate (50%), Malate (50%) | Succinate (50%), Malate (50%) |

*DX denotes diauxie and CoU signifies co-utilization.

## Supplementary References


1. Scott M, Gunderson CW, Mateescu EM, Zhang Z, Hwa T. Interdependence of cell growth and gene expression: origins and consequences. *Science* **330**, 1099-1102 (2010).
2. Wortel MT, Peters H, Hulshof J, Teusink B, Bruggeman FJ. Metabolic states with maximal specific rate carry flux through an elementary flux mode. *FEBS J.* **281**, 1547-1555 (2014).
3. You C, *et al.* Coordination of bacterial proteome with metabolism by cyclic AMP signalling. *Nature* **500**, 301-306 (2013).
4. Hui S, *et al.* Quantitative proteomic analysis reveals a simple strategy of global resource allocation in bacteria. *Mol. Syst. Biol.* **11**, 784 (2015).
5. Lehninger AL, Nelson DL, Cox MM. *Lehninger principles of biochemistry*, 5th edn. W.H. Freeman (2008).
6. Neidhardt FC, Ingraham JL, Schaechter M. *Physiology of the bacterial cell: a molecular approach*. Sinauer Sunderland (1990).
7. Schönheit P, Buckel W, Martin WF. On the origin of heterotrophy. *Trends Microbiol.* **24**, 12-25 (2016).
8. Okano H, Hermsen R, Kochanowski K, Sauer U, Hwa T. Regulation of hierarchical and simultaneous carbon-substrate utilization by flux sensors in Esherichia coli. *Unpublished*.
9. Bennett BD, Kimball EH, Gao M, Osterhout R, Van Dien SJ, Rabinowitz JD. Absolute metabolite concentrations and implied enzyme active site occupancy in Escherichia coli. *Nat. Chem. Biol.* **5**, 593 (2009).
10. Park JO, *et al.* Metabolite concentrations, fluxes and free energies imply efficient enzyme usage. *Nat. Chem. Biol.* **12**, 482 (2016).
11. Escalante-Chong R, *et al.* Galactose metabolic genes in yeast respond to a ratio of galactose and glucose. *Proc. Natl. Acad. Sci. U.S.A.* **112**, 1636-1641 (2015).
12. Phillips R, Milo R. A feeling for the numbers in biology. *Proc. Natl. Acad. Sci. U.S.A.* **106**, 21465-21471 (2009).
13. FARMER IS, JONES CW. The energetics of Escherichia coli during aerobic growth in continuous culture. *FEBS J.* **67**, 115-122 (1976).
14. Hempfling WP, Mainzer SE. Effects of varying the carbon source limiting growth on yield and maintenance characteristics of Escherichia coli in continuous culture. *J. Bacteriol.* **123**, 1076-1087 (1975).
15. Qian H. Phosphorylation energy hypothesis: open chemical systems and their biological functions. *Annu. Rev. Phys. Chem.* **58**, 113-142 (2007).
16. Tsai C. Spontaneous decarboxylation of oxalacetic acid. *Can. J. Chem.* **45**, 873-880

(1967).
17. Basan M, *et al.* A universal tradeoff between bacterial growth and adaptation. *Unpublished*.
18. Peyraud R, Kiefer P, Christen P, Portais J-C, Vorholt JA. Co-consumption of methanol and succinate by Methylobacterium extorquens AM1. *PLoS One* **7**, e48271 (2012).
19. Segel IH. *Enzyme kinetics: behavior and analysis of rapid equilibrium and steady state enzyme systems*. Wiley: New York (1993).
20. QIAN H. Open-system nonequilibrium steady state: Statistical thermodynamics, fluctuations, and chemical oscillations. *J. Phys. Chem. B* **110**, 15063-15074 (2006).
21. Liu X, *et al.* Reliable cell cycle commitment in budding yeast is ensured by signal integration. *eLife* **4**, e03977 (2015).
22. Wang X, Liu Y-Y. Overcome Competitive Exclusion in Ecosystems. *arXiv*.180506002 (2018).
23. Müller S, Regensburger G, Steuer R. Enzyme allocation problems in kinetic metabolic networks: optimal solutions are elementary flux modes. *J. Theor. Biol.* **347**, 182-190 (2014).
24. Schuster S, Hilgetag C. On elementary flux modes in biochemical reaction systems at steady state. *J. Biol. Syst.* **2**, 165-182 (1994).
25. Postma PW, Lengeler JW, Jacobson GR. Phosphoenolpyruvate:carbohydrate phosphotransferase systems of bacteria. *Microbiol. Rev.* **57**, 543-594 (1993).
26. Zamboni N, Fendt S-M, Rühl M, Sauer U. 13 C-based metabolic flux analysis. *Nat. Protoc.* **4**, 878 (2009).
27. Dauner M, Sauer U. GC-MS Analysis of Amino Acids Rapidly Provides Rich Information for Isotopomer Balancing. *Biotechnol. Prog.* **16**, 642-649 (2000).
28. Miller BG, Raines RT. Identifying latent enzyme activities: substrate ambiguity within modern bacterial sugar kinases. *Biochemistry* **43**, 6387-6392 (2004).
29. Meyer D, Schneider-Fresenius C, Horlacher R, Peist R, Boos W. Molecular characterization of glucokinase from Escherichia coli K-12. *J. Bacteriol.* **179**, 1298-1306 (1997).
30. Lunin VV, Li Y, Schrag JD, Iannuzzi P, Cygler M, Matte A. Crystal structures of Escherichia coli ATP-dependent glucokinase and its complex with glucose. *J. Bacteriol.* **186**, 6915-6927 (2004).
31. Gao H, Chen Y, Leary JA. Kinetic measurements of phosphoglucose isomerase and phosphomannose isomerase by direct analysis of phosphorylated aldose–ketose isomers using tandem mass spectrometry. *Int. J. Mass Spectrom.* **240**, 291-299 (2005).

32. Schreyer R, Bock A. Phosphoglucose isomerase from Escherischia coli K 10: purification, properties and formation under aerobic and anaerobic condition. *Arch. Microbiol.* **127**, 289-298 (1980).

33. Kotlarz D, Buc H. Phosphofructokinases from Escherichia coli. *Methods Enzymol.* **90**, 60-70 (1982).

34. Kotlarz D, Buc H. Two Escherichia coli fructose-6-phosphate kinases. Preparative purification, oligomeric structure and immunological studies. *Biochim. Biophys. Acta* **484**, 35-48 (1977).

35. Iancu CV, Mukund S, Fromm HJ, Honzatko RB. R-state AMP complex reveals initial steps of the quaternary transition of fructose-1,6-bisphosphatase. *J. Biol. Chem.* **280**, 19737-19745 (2005).

36. Brown G, *et al.* Structural and biochemical characterization of the type II fructose-1,6-bisphosphatase GlpX from Escherichia coli. *J. Biol. Chem.* **284**, 3784-3792 (2009).

37. Hao J, Berry A. A thermostable variant of fructose bisphosphate aldolase constructed by directed evolution also shows increased stability in organic solvents. *Protein Eng. Des. Sel.* **17**, 689-697 (2004).

38. Cooper SJ, *et al.* The crystal structure of a class II fructose-1,6-bisphosphate aldolase shows a novel binuclear metal-binding active site embedded in a familiar fold. *Structure* **4**, 1303-1315 (1996).

39. Straus D, Raines R, Kawashima E, Knowles JR, Gilbert W. Active site of triosephosphate isomerase: in vitro mutagenesis and characterization of an altered enzyme. *Proc. Natl. Acad. Sci. U.S.A.* **82**, 2272-2276 (1985).

40. Pichersky E, Gottlieb LD, Hess JF. Nucleotide sequence of the triose phosphate isomerase gene of Escherichia coli. *Mol. Gen. Genet.* **195**, 314-320 (1984).

41. D'Alessio G, Josse J. Glyceraldehyde phosphate dehydrogenase, phosphoglycerate kinase, and phosphoglyceromutase of Escherichia coli. Simultaneous purification and physical properties. *J. Biol. Chem.* **246**, 4319-4325 (1971).

42. Albe KR, Butler MH, Wright BE. Cellular concentrations of enzymes and their substrates. *J. Theor. Biol.* **143**, 163-195 (1990).

43. Fifis T, Scopes RK. Purification of 3-phosphoglycerate kinase from diverse sources by affinity elution chromatography. *Biochem. J.* **175**, 311-319 (1978).

44. D'Alession G, Josse J. Phosphoglycerate kinase and phosphoglyceromutase from Escherichia coli. *Methods Enzymol.* **42**, 139-144 (1975).

45. Fraser HI, Kvaratskhelia M, White MF. The two analogous phosphoglycerate mutases of

Escherichia coli. *FEBS Lett.* **455**, 344-348 (1999).

46. Spring TG, Wold F. Enolase from Escherichia coli. *Methods Enzymol* **42**, 323-329 (1975).

47. Spring TG, Wold F. The purification and characterization of Escherichia coli enolase. *J. Biol. Chem.* **246**, 6797-6802 (1971).

48. Malcovati M, Valentini G. AMP- and fructose 1,6-bisphosphate-activated pyruvate kinases from Escherichia coli. *Methods Enzymol.* **90**, 170-179 (1982).

49. Balakrishnan A, Nemeria NS, Chakraborty S, Kakalis L, Jordan F. Determination of pre-steady-state rate constants on the Escherichia coli pyruvate dehydrogenase complex reveals that loop movement controls the rate-limiting step. *J. Am. Chem. Soc.* **134**, 18644-18655 (2012).

50. Faloona GR, Srere PA. Escherichia coli citrate synthase. Purification and the effect of potassium on some properties. *Biochemistry* **8**, 4497-4503 (1969).

51. Duckworth HW, *et al.* Enzyme-substrate complexes of allosteric citrate synthase: evidence for a novel intermediate in substrate binding. *Biochim. Biophys. Acta* **1834**, 2546-2553 (2013).

52. Hodges M, *et al.* An iron regulatory-like protein expressed in Plasmodium falciparum displays aconitase activity. *Mol. Biochem. Parasitol.* **143**, 29-38 (2005).

53. Brock M, Maerker C, Schutz A, Volker U, Buckel W. Oxidation of propionate to pyruvate in Escherichia coli. Involvement of methylcitrate dehydratase and aconitase. *Eur. J. Biochem.* **269**, 6184-6194 (2002).

54. Hy M, Reeves HC. NADP+-specific isocitrate dehydrogenase of Excherichia coli. III. Two-step purification employing affinity chromatography. *Biochim. Biophys. Acta* **445**, 280-285 (1976).

55. Vasquez B, Reeves HC. NADP-specific isocitrate dehydrogenase of Escherichia coli. IV. Purification by chromatography on Affi-Gel Blue. *Biochim. Biophys. Acta* **578**, 31-40 (1979).

56. Waskiewicz DE, Hammes GG. Elementary steps in the reaction mechanism of the alpha-ketoglutarate dehydrogenase multienzyme complex from Escherichia coli: kinetics of succinylation and desuccinylation. *Biochemistry* **23**, 3136-3143 (1984).

57. Pettit FH, *et al.* α-Keto Acid Dehydrogenase Complexes XIX. subunit structure of the escherichia coli α-ketoglutarate dehydrogenase complex. *J. Biol. Chem.* **248**, 5282-5290 (1973).

58. Gibson J, Upper CD, Gunsalus IC. Succinyl coenzyme A synthetase from Escherichia coli. I. Purification and properties. *J. Biol. Chem.* **242**, 2474-2477 (1967).

59. Maklashina E, *et al.* Fumarate reductase and succinate oxidase activity of Escherichia

coli complex II homologs are perturbed differently by mutation of the flavin binding domain. *J. Biol. Chem.* **281**, 11357-11365 (2006).

60. Kim I, Bragg P. Some Properties of the Succinate Dehydrogenase of Escherichia coii. *Can. J. Biochem.* **49**, 1098-1104 (1971).

61. Weiner JH, Dickie P. Fumarate reductase of Escherichia coli. Elucidation of the covalent-flavin component. *J. Biol. Chem.* **254**, 8590-8593 (1979).

62. Estevez M, Skarda J, Spencer J, Banaszak L, Weaver TM. X-ray crystallographic and kinetic correlation of a clinically observed human fumarase mutation. *Protein Sci.* **11**, 1552-1557 (2002).

63. Woods SA, Schwartzbach SD, Guest JR. Two biochemically distinct classes of fumarase in Escherichia coli. *Biochim. Biophys. Acta* **954**, 14-26 (1988).

64. Murphey WH, Kitto GB. Malate dehydrogenase from Escherichia coli. *Methods Enzymol.* **13**, 145-147 (1969).

65. Kai Y, *et al.* Three-dimensional structure of phosphoenolpyruvate carboxylase: a proposed mechanism for allosteric inhibition. *Proc. Natl. Acad. Sci. U.S.A.* **96**, 823-828 (1999).

66. Smith TE. Escherichia coli phosphoenolpyruvate carboxylase. Physical and chemical properties. *J. Biol. Chem.* **246**, 4234-4241 (1971).

67. Goldie H, Medina V. Physical and genetic analysis of the phosphoenolpyruvate carboxykinase (pckA) locus from Escherichia coli K12. *Mol. Gen. Genet.* **220**, 191-196 (1990).

68. Tari LW, Matte A, Pugazhenthi U, Goldie H, Delbaere LT. Snapshot of an enzyme reaction intermediate in the structure of the ATP-Mg2+-oxalate ternary complex of Escherichia coli PEP carboxykinase. *Nat. Struct. Biol.* **3**, 355-363 (1996).

69. Goldie A, Sanwal BD. Allosteric control by calcium and mechanism of desensitization of phosphoenolpyruvate carboxykinase of Escherichia coli. *J. Biol. Chem.* **255**, 1399-1405 (1980).

70. Bologna FP, Andreo CS, Drincovich MF. Escherichia coli malic enzymes: two isoforms with substantial differences in kinetic properties, metabolic regulation, and structure. *J. Bacteriol.* **189**, 5937-5946 (2007).

71. Milne JA, Cook RA. Role of metal cofactors in enzyme regulation. Differences in the regulatory properties of the Escherichia coli nicotinamide adenine dinucleotide specific malic enzyme depending on whether Mg2+ or Mn2+ serves as divalent cation. *Biochemistry* **18**, 3604-3610 (1979).

72. Berman KM, Cohn M. Phosphoenolpyruvate synthetase of Escherichia coli. Purification,

some properties, and the role of divalent metal ions. *J. Biol. Chem.* **245**, 5309-5318 (1970).

73. Garcia-Alles LF, Zahn A, Erni B. Sugar recognition by the glucose and mannose permeases of Escherichia coli. Steady-state kinetics and inhibition studies. *Biochemistry* **41**, 10077-10086 (2002).

74. Lanz R, Erni B. The glucose transporter of the Escherichia coli phosphotransferase system. Mutant analysis of the invariant arginines, histidines, and domain linker. *J. Biol. Chem.* **273**, 12239-12243 (1998).

75. Ye L, Berden JA, van Dam K, Kruckeberg AL. Expression and activity of the Hxt7 high-affinity hexose transporter of Saccharomyces cerevisiae. *Yeast* **18**, 1257-1267 (2001).

76. Kornberg HL. Fructose transport by Escherichia coli. *Philos. Trans. Royal Soc. B* **326**, 505-513 (1990).

77. Buchel DE, Gronenborn B, Muller-Hill B. Sequence of the lactose permease gene. *Nature* **283**, 541-545 (1980).

78. Wright JK, Overath P. Purification of the lactose:H+ carrier of Escherichia coli and characterization of galactoside binding and transport. *Eur. J. Biochem.* **138**, 497-508 (1984).

79. Juers DH, Matthews BW, Huber RE. LacZ β-galactosidase: Structure and function of an enzyme of historical and molecular biological importance. *Protein Sci.* **21**, 1792-1807 (2012).

80. Jacobson R, Zhang X-J, DuBose R, Matthews B. Three-dimensional structure of β-galactosidase from E. coli. *Nature* **369**, 761 (1994).

81. Voegele RT, Sweet GD, Boos W. Glycerol kinase of Escherichia coli is activated by interaction with the glycerol facilitator. *J. Bacteriol.* **175**, 1087-1094 (1993).

82. Sweet G, *et al.* Glycerol facilitator of Escherichia coli: cloning of glpF and identification of the glpF product. *J. Bacteriol.* **172**, 424-430 (1990).

83. Milo R, Phillips R. *Cell biology by the numbers*. Garland Science (2015).

84. Thorner JW. Glycerol kinase. *Methods Enzymol.* **42**, 148-156 (1974).

85. Thorner JW, Paulus H. Composition and subunit structure of glycerol kinase from Escherichia coli. *J. Biol. Chem.* **246**, 3885-3894 (1971).

86. Schryvers A, Weiner JH. The anaerobic sn-glycerol-3-phosphate dehydrogenase of Escherichia coli. Purification and characterization. *J. Biol. Chem.* **256**, 9959-9965 (1981).

87. Poole RC, Halestrap AP. N-terminal protein sequence analysis of the rabbit erythrocyte lactate transporter suggests identity with the cloned monocarboxylate transport protein

MCT1. *Biochem. J.* **303** 755-759 (1994).

88. Halestrap AP. The monocarboxylate transporter family--Structure and functional characterization. *IUBMB Life* **64**, 1-9 (2012).

89. Kreth J, Lengeler JW, Jahreis K. Characterization of pyruvate uptake in Escherichia coli K-12. *PloS one* **8**, e67125 (2013).

90. Oliver DJ, Walker GH. Characterization of the transport of oxaloacetate by pea leaf mitochondria. *Plant Physiol.* **76**, 409-413 (1984).

91. Palmieri L, *et al.* Identification of the yeast mitochondrial transporter for oxaloacetate and sulfate. *J. Biol. Chem.* **274**, 22184-22190 (1999).

92. Karinou E, Compton EL, Morel M, Javelle A. The Escherichia coli SLC26 homologue YchM (DauA) is a C4-dicarboxylic acid transporter. *Mol. Microbiol.* **87**, 623-640 (2013).

93. Lo TC, Rayman MK, Sanwal BD. Transport of succinate in Escherichia coli I. Biochemical and genetic studies of transport in whole cells. *J. Biol. Chem.* **247**, 6323-6331 (1972).

94. Rayman MK, Lo TC, Sanwal BD. Transport of succinate in Escherichia coli II. Characteristics of uptake and energy coupling with transport in membrane preparations. *J. Biol. Chem.* **247**, 6332-6339 (1972).

95. Kleefeld A, Ackermann B, Bauer J, Krämer J, Unden G. The fumarate/succinate antiporter DcuB of Escherichia coli is a bifunctional protein with sites for regulation of DcuS dependent gene expression. *J. Biol. Chem.* **284**, 265-275 (2009).

96. Towbin BD, Korem Y, Bren A, Doron S, Sorek R, Alon U. Optimality and sub-optimality in a bacterial growth law. *Nat. Commun.* **8**, 14123 (2017).

97. Deutscher J, Francke C, Postma PW. How phosphotransferase system-related protein phosphorylation regulates carbohydrate metabolism in bacteria. *Microbiol. Mol. Biol. Rev.* **70**, 939-1031 (2006).
